# JAZ2/JAZ4-MYC2.1 module mediates MeJA-induced alleviation of chilling injury in peach fruit (*Prunus persica*)

**DOI:** 10.1093/hr/uhaf295

**Published:** 2025-11-03

**Authors:** Ang Li, Hongmei Wang, Akhi Badrunnesa, Junren Meng, Yuan Gao, Shihang Sun, Liang Niu, Lei Pan, Wenyi Duan, Guochao Cui, Zhiqiang Wang, Wenfang Zeng

**Affiliations:** National Key Laboratory for Germplasm Innovation & Utilization of Horticultural Crops, Zhengzhou Fruit Research Institute, Chinese Academy of Agricultural Sciences, Zhengzhou 450009, China; Zhongyuan Research Center, Chinese Academy of Agricultural Sciences, Xinxiang 453500, China; National Key Laboratory for Germplasm Innovation & Utilization of Horticultural Crops, Zhengzhou Fruit Research Institute, Chinese Academy of Agricultural Sciences, Zhengzhou 450009, China; National Key Laboratory for Germplasm Innovation & Utilization of Horticultural Crops, Zhengzhou Fruit Research Institute, Chinese Academy of Agricultural Sciences, Zhengzhou 450009, China; Zhongyuan Research Center, Chinese Academy of Agricultural Sciences, Xinxiang 453500, China; National Key Laboratory for Germplasm Innovation & Utilization of Horticultural Crops, Zhengzhou Fruit Research Institute, Chinese Academy of Agricultural Sciences, Zhengzhou 450009, China; Zhongyuan Research Center, Chinese Academy of Agricultural Sciences, Xinxiang 453500, China; National Key Laboratory for Germplasm Innovation & Utilization of Horticultural Crops, Zhengzhou Fruit Research Institute, Chinese Academy of Agricultural Sciences, Zhengzhou 450009, China; Zhongyuan Research Center, Chinese Academy of Agricultural Sciences, Xinxiang 453500, China; National Key Laboratory for Germplasm Innovation & Utilization of Horticultural Crops, Zhengzhou Fruit Research Institute, Chinese Academy of Agricultural Sciences, Zhengzhou 450009, China; Zhongyuan Research Center, Chinese Academy of Agricultural Sciences, Xinxiang 453500, China; National Key Laboratory for Germplasm Innovation & Utilization of Horticultural Crops, Zhengzhou Fruit Research Institute, Chinese Academy of Agricultural Sciences, Zhengzhou 450009, China; National Key Laboratory for Germplasm Innovation & Utilization of Horticultural Crops, Zhengzhou Fruit Research Institute, Chinese Academy of Agricultural Sciences, Zhengzhou 450009, China; Zhongyuan Research Center, Chinese Academy of Agricultural Sciences, Xinxiang 453500, China; National Key Laboratory for Germplasm Innovation & Utilization of Horticultural Crops, Zhengzhou Fruit Research Institute, Chinese Academy of Agricultural Sciences, Zhengzhou 450009, China; Zhongyuan Research Center, Chinese Academy of Agricultural Sciences, Xinxiang 453500, China; National Key Laboratory for Germplasm Innovation & Utilization of Horticultural Crops, Zhengzhou Fruit Research Institute, Chinese Academy of Agricultural Sciences, Zhengzhou 450009, China; National Key Laboratory for Germplasm Innovation & Utilization of Horticultural Crops, Zhengzhou Fruit Research Institute, Chinese Academy of Agricultural Sciences, Zhengzhou 450009, China; National Key Laboratory for Germplasm Innovation & Utilization of Horticultural Crops, Zhengzhou Fruit Research Institute, Chinese Academy of Agricultural Sciences, Zhengzhou 450009, China; Zhongyuan Research Center, Chinese Academy of Agricultural Sciences, Xinxiang 453500, China

## Abstract

Methyl jasmonate (MeJA) has emerged as a promising agent for mitigating chilling injury (CI) in peach fruit (*Prunus persica*); however, the molecular mechanisms underlying the role of MYC2, a key transcriptional regulator of jasmonic acid (JA) signaling, in mediating cold adaptation remain largely unexplored. In this study, we demonstrated that MeJA treatment effectively alleviated CI in peach fruit, accompanied by enhanced ethylene biosynthesis, elevated accumulation of polyphenols and flavonoids, and a marked reduction in reactive oxygen species levels. Using DNA affinity purification sequencing and transactivation assays, we identified PpMYC2.1 as a central regulator that directly activates key genes involved in ethylene-mediated fruit softening (*PpIAA1*, *PpHB.G7*, *PpERF61*, *PpPL1*, *PpPG2*, and *PpXTH2*) and phenylpropanoid metabolism (*PpPAL1*, *Pp4CL*, *PpCHI3*, and *PpCHS*). Stable overexpression of *PpMYC2.1* in tomato (*Solanum lycopersicum*) significantly enhanced fruit tolerance to cold stress. Meanwhile, transient overexpression or silencing in peach fruit upregulated or downregulated the expression of its target genes, confirming its positive regulatory role in cold stress response. Mechanistically, MeJA downregulated the expression of transcriptional repressors *PpJAZ2* and *PpJAZ4*, thereby alleviating their suppression of PpMYC2.1-mediated transactivation. Collectively, these findings reveal a previously uncharacterized JA-responsive transcriptional module, PpJAZ2/4-PpMYC2.1, that orchestrates cold stress adaptation in peach fruit, offering novel insights into postharvest preservation strategies for climacteric fruit.

## Introduction

Peach (*Prunus persica*), a popular fleshy fruit, is favored by consumers due to its appealing appearance, rich nutritional value, and delightful taste, securing its important position in the market [1]. Being a typical climacteric fruit, it is particularly subject to postharvest decay and quality deterioration [2]. Although cold storage is commonly employed to extend its shelf life, prolonged exposure to low temperatures can result in chilling injury (CI), a physiological disorder. This disorder is mainly reflected in symptoms, such as surface pitting, internal browning (IB), impaired ripening, and diminished flavor compound accumulation [3, 4]. Among CI symptoms, IB is particularly detrimental, primarily resulting from the enzymatic oxidation of intracellular phenolic compounds into quinones by polyphenol oxidase (PPO) and peroxidase (POD), which subsequently polymerize into brown pigments causing discoloration and degradation of internal fruit tissues [5]. These CI-related issues significantly compromise fruit marketability and consumer acceptance. Consequently, it is imperative to establish scientifically sound and effective postharvest strategies that can alleviate CI symptoms and preserve the quality of peach fruit during storage.

Methyl jasmonate (MeJA), a signaling molecule within plant, is essential for numerous physiological processes, including regulating intracellular signal transduction, plant growth, and coordinating responses to diverse environmental stresses [6]. The post-harvest application of MeJA has been widely proven to significantly enhance the cold resistance of various fruit, including peach [7], tomato [8], mango [9], loquat [10], and kiwifruit [11]. In peach fruit, the alleviation of CI symptoms by MeJA has been associated with multiple physiological processes, including the enhanced biosynthesis of ethylene and jasmonic acid (JA) [12], increased antioxidant capacity [13], accumulation of sucrose and aromatic compounds [14, 15], as well as higher unsaturated fatty acid content [7]. In addition, in other fruits, the mitigation of CI symptoms by MeJA involves the regulation of phenylpropanoid metabolism, polyamine synthesis, and the AsA-GSH cycle [16–18]. While the physiological effects of MeJA in mitigating CI have been thoroughly studied, the molecular mechanism—particularly the regulatory networks and signal transduction pathways underlying these responses—remain largely unexplored.

Previous research has established that MYC2 acts as a central regulatory node within the JA signaling network [19]. It demonstrates a strong and selective interaction with the G-box (5′-CANNTG-3′) cis-acting element, orchestrating the transcriptional activation of numerous genes that respond to MeJA [20]. Recent research has underscored the pivotal role of MYC2 in mediating MeJA-induced cold tolerance in fruit. For instance, in banana, MaMYC2 interacts with MaICE1 to regulate cold tolerance responses triggered by MeJA treatment [21]. In addition, SlMYC2 from tomato promotes fruit chilling resistance by directly regulating the expression of genes associated with polyamine biosynthesis—such as *SlARG1*, *SlARG2*, *SlADC*, and *SlODC*—thereby stabilizing cell membranes under cold stress [16]. Furthermore, SlMYC2 forms a regulatory module with SlMYB12 to synergistically activate the flavonoid biosynthesis pathway, leading to significantly increased antioxidant capacity of tomato fruit [22]. Beyond its role in JA signaling, MYC2 also regulates ethylene biosynthesis, a process vital for cold stress responses. In apple, MdMYC2 directly binds to the promoters of key ethylene biosynthesis genes, *MdACS1* and *MdACO1*, to enhance their expression. Furthermore, it indirectly regulates *MdACS1* transcription by increasing the activity of MdERF3, further contributing to ethylene biosynthesis [23]. These results underscore the integrative role of MYC2 in coordinating JA and ethylene signaling during cold stress adaptation. However, despite its central role in other fruit species, the transcriptional regulatory mechanisms by which MYC2 mediates MeJA-induced cold tolerance in peach fruit remain largely unclear.

Jasmonate ZIM domain (JAZ) proteins serve as core transcriptional repressors of JA signaling in plants, acting as key modulators of growth, development, and stress responses. Under normal conditions, JAZ proteins suppress JA-responsive gene expression by directly binding to MYC, thereby negatively regulating JA signaling transduction [24]. However, upon JA accumulation, JAZ proteins are recognized by the SCFCOI1 ubiquitin ligase complex, ubiquitinated, and subsequently degraded via the 26S proteasome pathway [25]. This degradation releases the transcriptional repression imposed on MYC2 and other downstream transcription factors (TFs), thereby enabling the activation of JA-responsive genes. Recent studies have revealed species-specific regulation by JAZ proteins in plant cold stress adaptation. For example, in *Camellia sinensis*, JA-mediated degradation of CsJAZ12 facilitates the formation of the MYC2-MYB transcriptional complex, which promotes linalool biosynthesis and enhances cold tolerance [26]. In apple, MdJAZ1 and MdJAZ2 negatively impact cold tolerance through the disruption of brassinosteroid signaling: they inhibit MdBIM1-mediated activation of MdCBF1, which interferes with the formation of the MdBIM1–MdCBF2 complex, and they also suppress the transcriptional activity of MdBBX37 on MdCBF1 and MdCBF4 [27, 28]. Despite these advances, the specific JAZ regulators mediating cold tolerance in peach fruit induced by MeJA remain uncharacterized.

This study aims to determine the regulatory role of exogenous MeJA treatment in enhancing the cold stress tolerance of peach fruit during low-temperature postharvest storage. Therefore, we employed RNA sequencing (RNA-seq) to analyze changes in gene expression in peach fruit following MeJA treatment. By integrating DNA affinity purification sequencing (DAP-seq) data for PpMYC2.1, we identified genes directly targeted and activated by PpMYC2.1, including those involved in ethylene biosynthesis, cell wall degradation, and phenylpropanoid metabolism. Additionally, molecular interaction experiments were conducted to screen for PpJAZ proteins upstream of PpMYC2.1. These findings unveil a previously uncharacterized transcriptional cascade in which MeJA modulates the PpJAZ2/4–PpMYC2.1 module to coordinate JA–ethylene crosstalk during cold adaptation. This work not only provides novel insights into the regulatory circuitry underpinning fruit cold stress tolerance but also offering promising molecular targets for optimizing postharvest storage and cold chain logistics in climacteric fruit.

## Results

### MeJA treatment enhance cold tolerance of peach fruit

**Figure 1:**
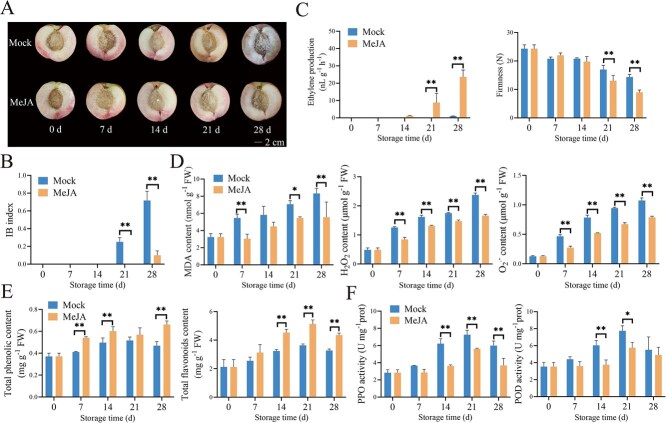
Exogenous MeJA treatment alleviates CI symptoms in peach fruit. (A) Phenotypic comparison of CI development in control and MeJA-treated peach fruit after cold storage. Scale bar = 2 cm. (B) Effects of MeJA treatment on IB index. (C) Effects of MeJA treatment on ethylene biosynthesis and fruit firmness. (D) Effects of MeJA treatment on MDA, O_2_^·−^, and H_2_O_2_ levels. (E) The impact of MeJA application on total phenolic and flavonoid levels. (F) The impact of MeJA application on the enzymatic activities of PPO and POD. Error bars represent means ± standard error (SE) (n = 3 biological replicates). Asterisks indicate significant differences as determined by Student’s *t*-test, with ^*^P < 0.05 and ^**^P < 0.01.

SH peach (*P. persica* cv. CP9), characterized by its inherent deficiency in ethylene biosynthesis, exhibits heightened susceptibility to CI, primarily manifested as an increased IB. To investigate the role of MeJA and its potential crosstalk with ethylene signaling in modulating CI during postharvest cold storage, peach fruits were treated with 1 mM MeJA. We found that the contents of JA and jasmonoyl-isoleucine (JA-Ile) in MeJA-treated peach fruit peaked on the 7 days of cold storage and then gradually decreased, and exhibited significantly higher contents compared to those in the control (mock group) fruit throughout the storage period (Fig. S1). As shown in Fig. 1A and B, control fruit exhibited initial symptoms of CI after 21 days of cold storage, with IB severity progressively intensifying over time. In contrast, MeJA treatment effectively suppressed the progression of IB. Ethylene biosynthesis patterns revealed notable differences between treatments during cold storage. The control group showed minimal ethylene production throughout the storage period, whereas MeJA-treated fruit initiated ethylene emission at 14 days post-treatment, reaching a 23-fold increase compared to control by day 28. This ethylene surge was accompanied by a noticeable reduction in fruit firmness (Fig. 1C). Biochemical analyses further demonstrated that although the levels of malondialdehyde (MDA), superoxide anion (O₂^·−^), and hydrogen peroxide (H₂O₂) increased over time in all samples, the accumulation rates were lower in MeJA-treated peach fruit compared to the control (Fig. 1D).

Enhancing the antioxidant defense system is a critical strategy for alleviating IB in postharvest fruit. Our findings showed that the treatment with MeJA significantly enhanced the accumulation of key antioxidant compounds, including total phenols and flavonoids, in comparison to the control (Fig. 1E). Meanwhile, it markedly reduced the enzymatic activity of oxidative stress-related enzymes, PPO and POD, thereby effectively limiting oxidative substrate degradation (Fig. 1F).

### PpMYC2.1 actively participates in MeJA-regulated cold tolerance in peach fruit

To comprehensively explore the transcriptional networks through which MeJA alleviates CI in peach fruit, RNA-seq was performed. In total, 27 RNA-seq libraries were constructed, each generated approximately 42.78 million clean reads, with a Q30 score exceeding 97.23% and 96.07% of the reads uniquely aligned to the *P. persica* genome, indicating high accuracy and reliability (Table S1). A total of 3039, 4936, 7027, and 6452 differentially expressed genes (DEGs) were detected between the MeJA-treated and control groups in peach fruit at 7, 14, 21, and 28 days, respectively (Fig. S2A). Furthermore, the maximum number of uniquely DEGs was identified during the development stage of the CI, suggesting that this period is critical for transcriptomic reprogramming (Fig. S2B). These findings indicate that MeJA treatment significantly regulates gene expression in peach fruit, particularly during the early phase of CI initiation. Based on the physiological evidence supporting MeJA-mediated alleviation of CI, several DEGs associated with ethylene biosynthesis and signaling, polyphenol metabolism, cell wall degradation, and JA signaling pathway were identified (Fig. S2C–F). These genes are expected to be crucial components of the regulatory network underlying the MeJA-induced mitigation of CI.

**Figure 2:**
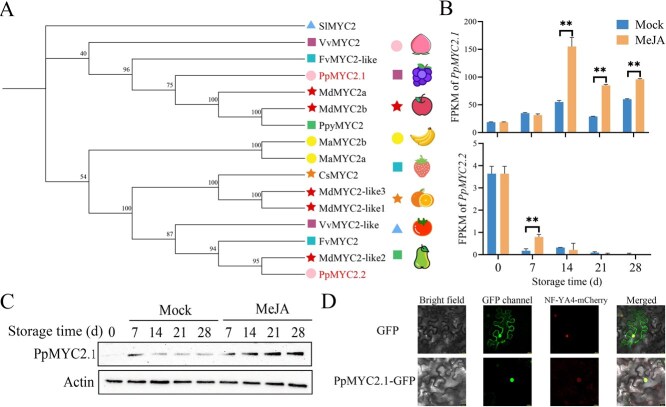
PpMYC2.1 plays a role in MeJA-mediated JA signal pathway in peach fruit. (A) Phylogenetic analysis of PpMYC2.1 and PpMYC2.2. The phylogenetic tree was generated utilizing Tbtools [29], with bootstrap values from 1000 replicates displayed at each branch. Sl, *Solanum lycopersicum*; Md, *Malus domestica*; Fv, *Fragaria vesca*; Ppy, *Pyrus pyrifolia*; Vv, *Vitis vinifera*; Ma, *Musa acuminata*; Cs, *Citrus sinensis*. (B) Gene expression profiles of *PpMYC2.1* and *PpMYC2.2* in peach fruit subjected to MeJA treatment during cold storage. (C) Western blot analysis demonstrating the accumulation of PpMYC2.1 protein in peach fruit subjected to MeJA treatment during cold storage. (D) Subcellular localization of PpMYC2.1-GFP expressed in *Nicotiana benthamiana* leaf cells. NLS-mCherry served as a nuclear marker and exhibited complete overlap with the GFP fluorescence observed in the nucleus. Scale bar = 20 μm. Error bars represent means ± SE (*n* = 3 biological replicates). Asterisks indicate significant differences as determined by Student’s *t*-test, with ^*^P < 0.05 and ^**^P < 0.01.

MYC2 TF functions as a ‘molecular switch’ in the JA signaling pathway. In the peach genome, two MYC2 homologs were identified: *PpMYC2.1* and *PpMYC2.2*. Phylogenetic investigation indicated that PpMYC2.1 is most closely related to MdMYC2a and MdMYC2b from apple (*Malus domestica*) and PpyMYC2 from pear (*Pyrus pyrifolia*), whereas PpMYC2.2 shows higher similarity to MdMYC2-like2 from apple and FvMYC2 from strawberry (*Fragaria vesca*) (Fig. 2A), suggesting evolutionary divergence in the regulatory functions of the two homologs. Tissue-specific expression profiling further revealed that *PpMYC2.2* expression is restricted to roots and stems, while *PpMYC2.1* is ubiquitously expressed across all examined tissues (Fig. S3), indicating that PpMYC2.1 may play a broader role in systemic JA-mediated responses.

Under MeJA treatment during cold storage, *PpMYC2.1* expression was significantly upregulated, beginning at 14 days, whereas *PpMYC2.2* exhibited a transient induction limited to 7 days, followed by a decline to undetectable levels (Fig. 2B). Further analysis of correlations indicated a positive association between *PpMYC2.1* expression and ethylene production, as well as total polyphenol and flavonoid accumulation, with correlation coefficients (*r*) of 0.4, 0.72, and 0.63, respectively. In contrast, *PpMYC2.2* exhibited negative correlations with these parameters, with *r* values of −0.27, −0.6, and −0.57, respectively (Fig. S4). Immunoblot analysis confirmed the MeJA-induced accumulation of PpMYC2.1 protein (Fig. 2C). Additionally, transient expression of a PpMYC2.1–GFP fusion construct in *Nicotiana benthamiana* leaves revealed exclusive nuclear localization, as demonstrated by colocalization with the nuclear marker NLS-mCherry (Fig. 2D). Collectively, these findings identify PpMYC2.1 as an important regulator of MeJA-induced cold tolerance in peach fruit, acting through integrated regulation of ethylene signaling and antioxidant biosynthesis.

### Genome-wide identification of potential targets of PpMYC2.1

**Figure 3:**
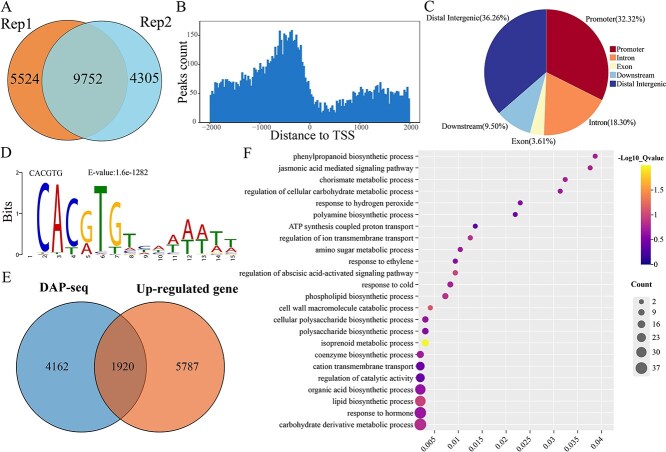
Integrated analysis of DAP-seq and RNA-seq data identified direct target genes regulated by PpMYC2.1. (A) The analysis of PpMYC2.1 binding peaks across two technical replicates. (B) Distribution of PpMYC2.1 binding sites relative to TSS, showing enrichment near the TSS. (C) Genomic distribution of PpMYC2.1 binding peaks. (D) Enriched binding motifs of PpMYC2.1 identified using HOMER software. (E) Overlap between PpMYC2.1-bound genes as identified by DAP-seq and upregulated genes detected by RNA-seq. (F) GO enrichment analysis of candidate direct target genes regulated by PpMYC2.1.

DAP-seq has become an effective method for profiling TF binding sites across the genome in a near-native genomic context [30, 31]. To elucidate the role of PpMYC2.1 in JA signaling transduction, we conducted DAP-seq to systematically characterize its cis-regulatory landscape. Quality metrics for sequencing, such as the count of high-quality reads and the rates of unique mappings for each library, are provided in Table S2. The two biological replicates showed high consistency, both identifying 9752 binding peaks (Data Set S1), which were considered high-confidence PpMYC2.1 binding regions (Fig. 3A). These peaks were primarily enriched within the 1000-bp upstream regions of transcription start site (TSS) (Fig. 3B). Analysis of peak distribution revealed that PpMYC2.1 binds to diverse genomic regions, including exons (3.61%), introns (18.3%), downstream regions (9.5%), intergenic regions (36.26%), and importantly, promoter regions (33.32%) (Fig. 3C). This distribution pattern supports the function of PpMYC2.1 as a TF that is involved in DNA-binding and transcriptional regulation. The most enriched motif identified was CAC[G/A]TG (Fig. 3D), which closely resembles the canonical G-box (5′-CACGTG-3′) and its variant (5′-CACATG-3′) [32, 33]. Additionally, PpMYC2.1 binding sites were uniformly distributed across all eight *P. persica* chromosomes, indicating no specific chromosomal bias in its binding pattern (Fig. S5).

To further elucidate the direct regulatory targets of PpMYC2.1, a comparative analysis was performed between the 6082 PpMYC2.1-bound genes identified in the DAP-seq dataset and the 7707 upregulated genes identified in the RNA-seq analysis. This integration revealed an overlap of 1920 genes, representing putative direct targets of PpMYC2.1 (Fig. 3E). Gene ontology (GO) enrichment analysis of these overlapping genes highlighted significant enrichment in several metabolic pathways, including ‘phenylpropanoid biosynthetic process’, ‘jasmonic acid-mediated signaling pathway’, ‘response to ethylene’, and ‘cell wall macromolecule catabolic process’ (Fig. 3F). Consistent with previously reported functions of MYC2 in fruit physiology, PpMYC2.1 was found to directly regulate key genes involved in ethylene biosynthesis, cell wall degradation, and phenylpropanoid metabolism, suggesting its central role in mediating MeJA-induced cold tolerance in peach fruit (Fig. S6).

### PpMYC2.1 directly regulates key TFs in the ethylene biosynthesis pathway

**Figure 4:**
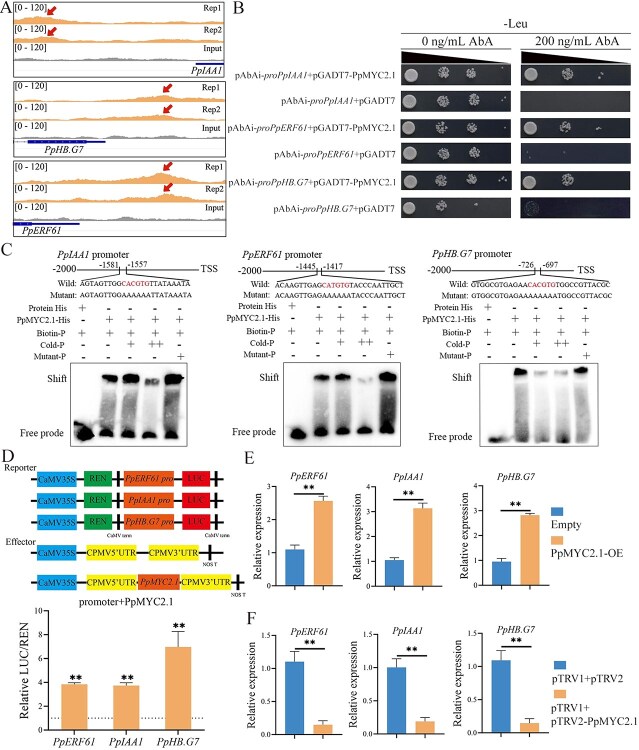
PpMYC2.1-mediated direct regulation of key TFs involved in ethylene biosynthesis in peach fruit. (A) DAP-seq analysis showing PpMYC2.1 binding sites and input control signals across the genomic loci of *PpIAA1*, *PpERF61*, and *PpHB.G7*. (B) Y1H demonstrating the binding of PpMYC2.1 to promoter fragments of *PpIAA1*, *PpERF61*, and *PpHB.G7* in yeast cells. In this experiment, pGADT7-PpMYC2.1 was used as the prey, while pAbAi constructs containing the promoter sequences (*pAbAi-proPpIAA1*, *pAbAi-proPpERF61*, and *pAbAi-proPpHB.G7*) served as the baits. Transformants were selected on synthetic dropout (SD)/-leucine (Leu) medium supplemented with AbA. (C) EMSA showing that PpMYC2.1 directly binds to cis-elements in the promoters of *PpIAA1*, *PpERF61*, and *PpHB.G7*. (D) DLR assay analyzing the transcriptional activation of PpMYC2.1 on *PpIAA1*, *PpERF61*, and *PpHB.G7*. Error bars represent means ± SE (n = 6 biological replicates). (E) Transient overexpression of *PpMYC2.1* in peach fruit significantly upregulated the expression of *PpIAA1*, *PpERF61*, and *PpHB.G7*. (F) Transient silencing of *PpMYC2.1* in peach fruit significantly downregulated the expression of *PpIAA1*, *PpERF61*, and *PpHB.G7*. Error bars represent means ± SE (*n* = 3 biological replicates). Asterisks indicate significant differences as determined by Student’s *t*-test, with ^*^P < 0.05 and ^**^P < 0.01.

Previous studies have demonstrated that exogenous ethylene application can alleviate the CI symptoms of peach fruit [34, 35], with *PpACS1* and *PpACO1* identified as key regulatory genes in ethylene biosynthesis [36]. However, DAP-seq analysis revealed that neither *PpACS1* nor *PpACO1* is direct targets of PpMYC2.1 (Data Set S1), suggesting that PpMYC2.1 may modulate ethylene biosynthesis through indirect mechanisms. Notably, *PpIAA1*, *PpERF61*, and *PpHB.G7* exhibited enriched binding sites in their promoter regions, indicating that these TFs may serve as intermediaries in PpMYC2.1-mediated regulation of the ethylene biosynthetic pathway (Fig. 4A). To validate the interaction between PpMYC2.1 and the promoters of its potential target genes, a yeast one-hybrid (Y1H) assay was conducted. In yeast grown on medium supplemented with aureobasidin A (AbA; 200 ng/ml), no basal reporter gene activity was detected in strains harboring only the promoters of *PpERF61*, *PpIAA1*, and *PpHB.G7*, as evidenced by the absence of growth in yeast cells (Fig. 4B). In contrast, co-expression of PpMYC2.1 with each of the target promoters resulted in robust growth on AbA-containing medium, indicating successful activation of the AbA resistance reporter gene (Fig. 4B). These findings indicate that PpMYC2.1 directly interacts with the promoter regions of *PpERF61*, *PpIAA1*, and *PpHB.G7* in yeast cells.

To further assess the direct binding capacity of PpMYC2.1 to cis-regulatory elements within target gene promoters, electrophoretic mobility shift assay (EMSA) were conducted using purified recombinant PpMYC2.1 protein. Biotin-labeled DNA probes, which contained the conserved binding motifs identified through DAP-seq, were incubated with the recombinant protein. The formation of protein–DNA complexes was evidenced by distinct mobility shifts for all tested promoter fragments of *PpERF61*, *PpIAA1*, and *PpHB.G7,* confirming the direct interaction between PpMYC2.1 and these promoter regions (Fig. 4C). The addition of cold probes (i.e. unlabeled competitor probes) progressively reduced the intensity of the shifted bands. In contrast, probes containing mutated binding motifs failed to compete effectively for the binding, demonstrating the sequence-specific binding of PpMYC2.1 (Fig. 4C). Furthermore, no mobility shift was detected when only the His-tag control protein was incubated with biotin-labeled probes (Fig. 4C). Taken together, these findings offer strong in vitro evidence that PpMYC2.1 specifically binds to the promoter regions of *PpERF61*, *PpIAA1*, and *PpHB.G7.*

Dual-luciferase reporter (DLR) assays were performed to evaluate the regulatory effect of PpMYC2.1 on its target gene promoters. The *PpMYC2.1* coding sequence, regulated by the CaMV 35S promoter, was utilized as the effector, while the LUC reporter gene, controlled by the promoter of each target gene, functioned as the reporter. The results revealed that PpMYC2.1 significantly activated the promoters of *PpIAA1* (3-fold), *PpERF61* (3-fold), and *PpHB.G7* (6-fold), thereby markedly enhancing LUC reporter gene expression (Fig. 4D). Due to the lack of a stable genetic transformation system suitable for peach, we conducted a transient overexpression and silencing of *PpMYC2.1* in the flesh of peach fruit to evaluate its regulatory impact on downstream target genes. Compared to the control group, both transcript and protein levels of PpMYC2.1 were significantly increased in the overexpressing fruit, while the silenced fruit exhibited a marked reduction in both (Fig. S7). This transient overexpression led to a marked upregulation of *PpIAA1*, *PpERF61,* and *PpHB.G7* expression, whereas silencing led to a reduction in their expression (Fig. 4E and F). These findings demonstrate that PpMYC2.1 can directly interact with the promoter regions of these target genes, thereby facilitating their transcriptional activation and modulating ethylene biosynthesis during low-temperature storage of peach fruit.

### PpMYC2.1 directly modulates core genes involved in cell wall degradation

**Figure 5:**
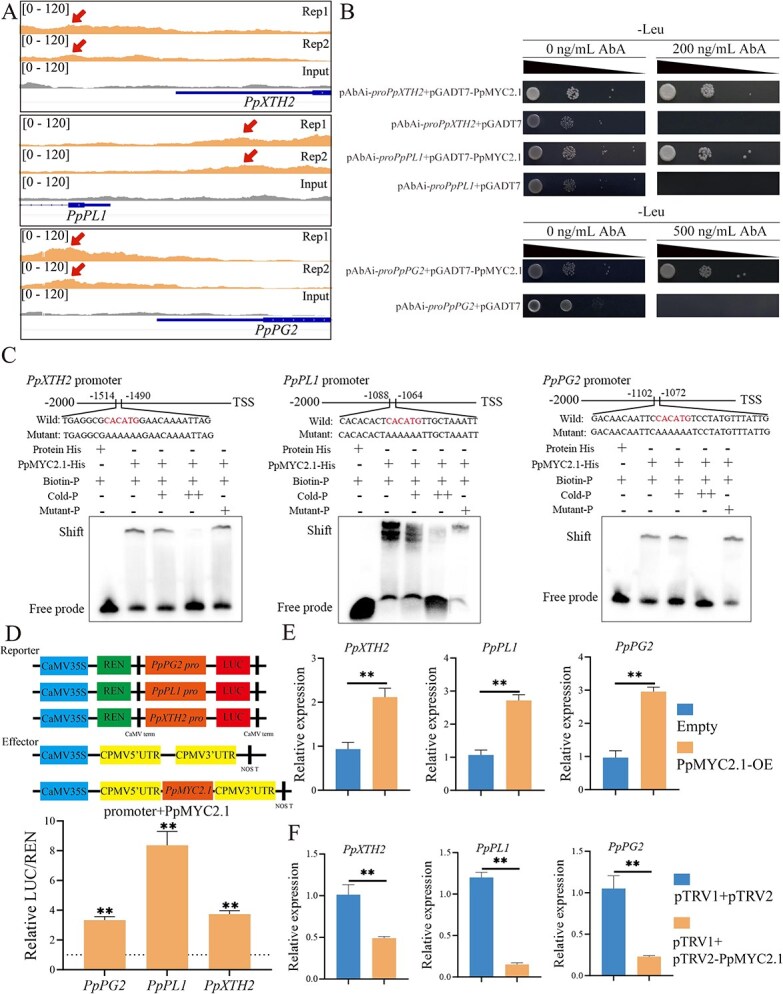
PpMYC2.1-mediated direct regulation of key genes associated with cell wall degradation in peach fruit. (A) DAP-seq analysis showing PpMYC2.1 binding sites and input control signals across the genomic loci of *PpXTH2*, *PpPL*1, and *PpPG2*. (B) Y1H assay demonstrating the interaction of PpMYC2.1 with the promoter regions of *PpXTH2*, *PpPL1*, and *PpPG2* in yeast cells. In this experiment, pGADT7-PpMYC2.1 was used as the prey, while *pAbAi-proPpXTH2*, *pAbAi-proPpPL1*, and *pAbAi-proPpPG2* served as the baits. Transformants were cultured on SD/-Leu medium supplemented with AbA to validate the interactions. (C) EMSA showing that PpMYC2.1 directly binds to specific sequence motifs within the promoters of *PpXTH2*, *PpPL1*, and *PpPG2*. (D) DLR assay analyzing the transcriptional activation of the *PpXTH2*, *PpPL1*, and *PpPG2* promoters by PpMYC2.1. Error bars represent means ± SE (*n* = 6 biological replicates). (E) Transient overexpression of *PpMYC2.1* in peach fruit significantly upregulated the expression levels of *PpXTH2*, *PpPL1*, and *PpPG2*. (F) Transient silencing of *PpMYC2.1* in peach fruit significantly downregulated the expression of *PpXTH2*, *PpPL1*, and *PpPG2*. Error bars represent means ± SE (*n* = 3 biological replicates). Asterisks indicate significant differences as determined by Student’s *t*-test, with ^*^P < 0.05 and ^**^P < 0.01.

By performing a combined analysis of DAP-seq and RNA-seq datasets, we determined that three cell wall degradation-related genes—*PpXTH2*, *PpPL1*, and *PpPG2*—are direct transcriptional targets of PpMYC2.1, with significant binding peaks detected in their promoter regions (Fig. 5A). Y1H analysis confirmed that PpMYC2.1 interacts with the promoters of these genes (Fig. 5B), while EMSA further validated its direct binding to these promoter regions in vitro (Fig. 5C). The DLR assay results demonstrated that PpMYC2.1 markedly enhanced the activity of the promoters of *PpPG2* (3-fold), *PpPL1* (8-fold), and *PpXTH2* (4-fold) (Fig. 5D). Transient overexpression in peach fruit flesh demonstrated that PpMYC2.1 activates the expression of *PpXTH2*, *PpPL1*, and *PpPG2* (Fig. 5E). Similarly, silencing of *PpMYC2.1* resulted in a marked decrease in the expression of these genes (Fig. 5F). These findings collectively indicate that PpMYC2.1 functions as a transcriptional enhancer of cell wall degradation genes, facilitating normal fruit softening, under low-temperature storage conditions.

### PpMYC2.1 directly regulates core genes involved in polyphenol and flavonoid biosynthesis

**Figure 6:**
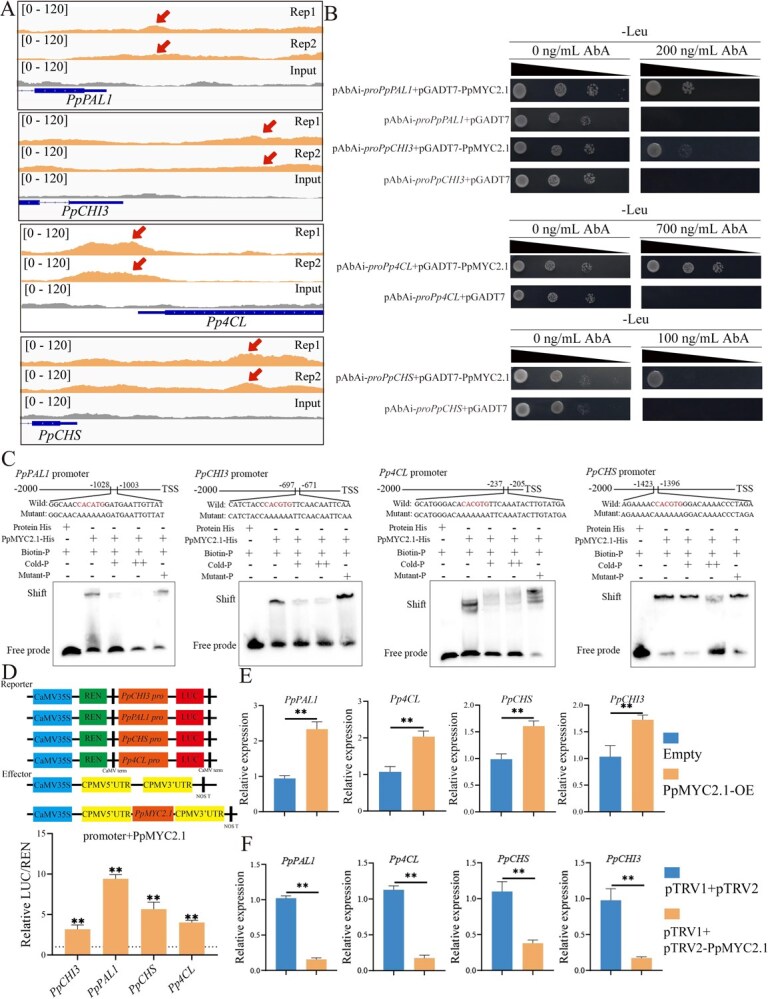
PpMYC2.1-mediated direct regulation of key genes associated with polyphenol and flavonoid biosynthesis in peach fruit. (A) DAP-seq analysis showing PpMYC2.1 binding sites and input control signals across the genomic loci of *PpPAL1*, *Pp4CL*, *PpCHS*, and *PpCHI3*. (B) Y1H assay demonstrating the interaction between PpMYC2.1 and the promoter regions of *PpPAL1*, *Pp4CL*, *PpCHS*, and *PpCHI3* in yeast cells. In this experiment, pGADT7-PpMYC2.1 was used as the prey, while *pAbAi-proPpPAL1*, *pAbAi-proPp4CL*, *pAbAi-proPpCHS*, and *pAbAi-proPpCHI3* served as the baits. Transformants were cultured on SD/-Leu medium supplemented with AbA to validate the interactions. (C) EMSA confirming that PpMYC2.1 directly binds to sequence motifs in the promoter regions of *PpPAL1*, *Pp4CL*, *PpCHS* and *PpCHI3*. (D) DLR assay assessing the transcriptional activation of the *PpPAL1*, *Pp4CL*, *PpCHS,* and *PpCHI3* promoters by PpMYC2.1. Error bars represent means ± SE (*n* = 6 biological replicates). (E) Transient overexpression of PpMYC2.1 in peach fruit significantly upregulated the expression levels of *PpPAL1*, *Pp4CL*, *PpCHS*, and *PpCHI3*. (F) Transient silencing of *PpMYC2.1* in peach fruit significantly downregulated the expression of *PpPAL1*, *Pp4CL*, *PpCHS,* and *PpCHI3*. Error bars represent means ± SE (*n* = 3 biological replicates). Asterisks indicate significant differences as determined by Student’s *t*-test, with ^*^P < 0.05 and ^**^P < 0.01.

Polyphenols and flavonoids, synthesized through the phenylpropanoid pathway, are key antioxidant compounds in plants and are closely linked to enhanced cold resistance in fruit [37]. DAP-seq analysis showed that PpMYC2.1 bound to the promoter regions of *PpPAL1*, *Pp4CL*, *PpCHI3*, and *PpCHS*, key genes in the phenylpropanoid pathway (Fig. 6A). Y1H assays confirmed that PpMYC2.1 bind to the promoters of these four genes in yeast cells (Fig. 6B). In addition, EMSA demonstrated that PpMYC2.1 specifically binds to the promoters of these genes in vitro (Fig. 6C). DLR assays showed that PpMYC2.1 activated the promoters of *PpCHI3* (3-fold), *PpPAL1* (8-fold), *PpCHS* (6-fold), and *Pp4CL* (4-fold) (Fig. 6D). Transient overexpression of *PpMYC2.1* in fruit flesh further revealed that it can activate the expression of *PpPAL1*, *Pp4CL*, *PpCHI3*, and *PpCHS* (Fig. 6E). Correspondingly, silencing *PpMYC2.1* suppressed the expression of these genes (Fig. 6F). These findings collectively suggest that PpMYC2.1 directly influences the transcription of genes involved in polyphenol and flavonoid biosynthesis, thereby improving the antioxidant capacity of peach fruit to withstand low-temperature stress.

### PpJAZ2/4 proteins participate in the JA signaling pathway through interaction with PpMYC2.1

The JAZ-MYC module constitutes a fundamental regulatory mechanism within the JA signaling, in which JAZ proteins function as repressors by directly binding to and inhibiting MYC TFs, thereby modulating downstream gene expression. In the peach genome, nine JAZ genes were identified (Fig. S8), all of which contain the conserved TIFY and Jas domains characteristic of the JAZ protein family (Fig. S9). Transcriptome profiling across diverse tissues and developmental stages demonstrated negligible expression of *PpJAZ6* and *PpJAZ8*, whereas *PpJAZ2* and *PpJAZ4* were grouped together in a distinct expression cluster, with the remaining JAZ members forming a separate cluster (Fig. S10). Similarly, during cold storage, *PpJAZ6* and *PpJAZ8* exhibited no detectable expression. Notably, MeJA treatment significantly upregulated the expression of *PpJAZ1*, *PpJAZ3*, *PpJAZ5*, *PpJAZ7*, and *PpJAZ10*, while the expression of *PpJAZ2* and *PpJAZ4* was significantly downregulated (Fig. S2F). Consistently, immunoblotting with specific antibodies against PpJAZ2 and PpJAZ4 showed that their protein levels decreased significantly after MeJA treatment at all time points (Fig. S11). Given this inverse expression pattern relative to MYC2 activity, PpJAZ2 and PpJAZ4 were selected as key candidates for further functional analysis.

**Figure 7:**
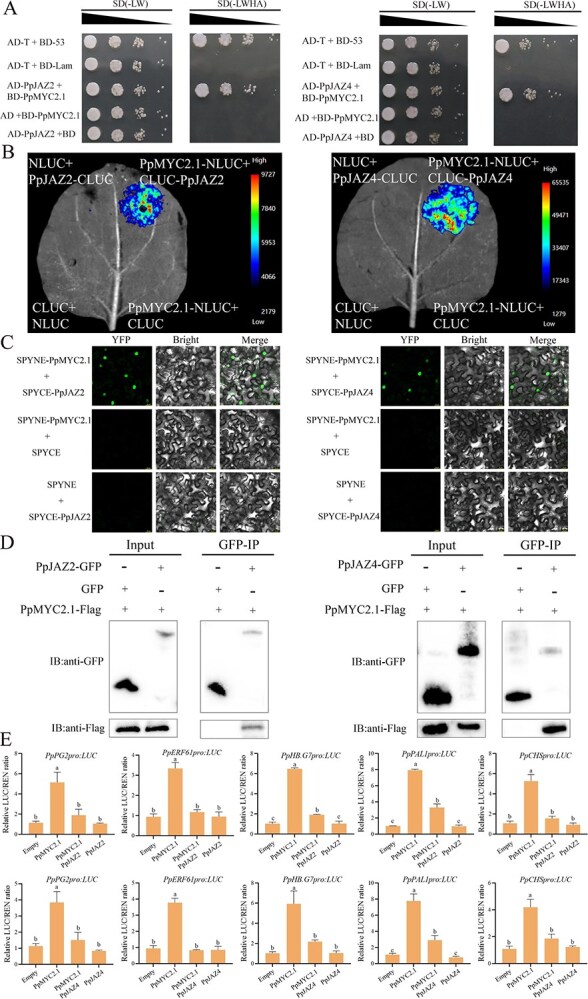
PpJAZ2/4 represses the transcriptional activity of PpMYC2.1 on its downstream target genes. (A) Y2H assay demonstrating the interaction between PpMYC2.1 and PpJAZ2/4. Empty vector served as negative control. (B) LCI assay demonstrating the interaction between PpMYC2.1 and PpJAZ2/4 in *N. benthamiana* leaves. Negative controls included PpMYC2.1-NLUC/CLUC, NLUC/CLUC-PpJAZ2/4, and NLUC/CLUC. (C) BiFC assay demonstrating the interaction between PpMYC2.1 and PpJAZ2/4. The SPYNE-PpMYC2.1 and SPYCE-PpJAZ2/4 were co-transformed in *N. benthamiana* leaves. The empty SPYNE or SPYCE constructs were used as negative controls. Scale bar = 20 μm. (D) In vivo Co-IP assay confirming the interaction between PpMYC2.1 and PpJAZ2/4. Co-expression of GFP and PpMYC2.1-Flag served as the negative control. (E) DLR assay demonstrating that PpJAZ2/4 suppresses PpMYC2.1-mediated activation of target promoters. Reporter plasmid of *PpPG2pro:LUC*, *PpERF61pro:LUC*, *PpHB.G7pro:LUC*, *PpPAL1pro:LUC*, and *PpCHSpro:LUC* promoters, and effector plasmids PpMYC2.1 or PpJAZ2/4 were co-expressed in *N. benthamiana* leaves. The empty vector + pro:LUC was normalized to 1 and served as a negative control. Data represent means ± SE (*n* = 6 biological replicates). Statistically significant differences were determined by one-way ANOVA followed by Tukey’s honestly significant difference (HSD) (*P* < 0.05) and are indicated by different lowercase letters.

To investigate the physical interaction between PpMYC2.1 and PpJAZ2/4, we performed a yeast two-hybrid (Y2H) analysis. We observed that yeast cells co-transformed with these constructs were able to grow on SD-tryptophan (Trp)/-Leu/-histidine (His)/-adenine (Ade) medium, indicating a direct interaction between PpJAZ2/4 and PpMYC2.1 proteins in yeast cells (Fig. 7A). Further studies have revealed that both PpJAZ2 and PpJAZ4 are localized in the nucleus, consistent withPpMYC2.1 (Fig. S12). In the luciferase complementation imaging (LCI) assay, reconstituted luciferase activity was detected when PpMYC2.1-NLUC was co-expressed with CLUC-PpJAZ2/4, whereas no signal was detected in the negative control combinations (Fig. 7B). The bimolecular fluorescence complementation (BiFC) assay results demonstrated that PpJAZ2/4 and PpMYC2.1 can form a heterodimer in nuclei (Fig. 7C). For the co-immunoprecipitation (Co-IP) assay, PpJAZ2/4-GFP successfully immunoprecipitated PpMYC2.1-Flag using an anti-GFP antibody, whereas no interaction was detected with the GFP control (Fig. 7D). Overall, these findings validate the interactions between PpJAZ2/4 and PpMYC2.1 in both in vitro and in vivo contexts.

Next, we employed a DLR assay to evaluate the effect of PpJAZ2/4 on PpMYC2.1-mediated transcriptional regulation of downstream target genes. Five randomly selected PpMYC2.1 target genes—*PpPG2*, *PpERF61*, *PpHB.G7*, *PpPAL1*, and *PpCHS*—were analyzed. As expected, PpMYC2.1 enhanced LUC activity driven by the promoters of these genes in comparison to the empty vector control. However, this transcriptional activation was attenuated upon co-expression with PpJAZ2 or PpJAZ4 (Fig. 7E). These results indicate that MeJA treatment significantly suppresses *PpJAZ2/4* expression, thereby relieving PpMYC2.1-mediated transcription of downstream genes.

### Overexpression of *PpMYC2.1* increases cold tolerance in transgenic tomato

**Figure 8:**
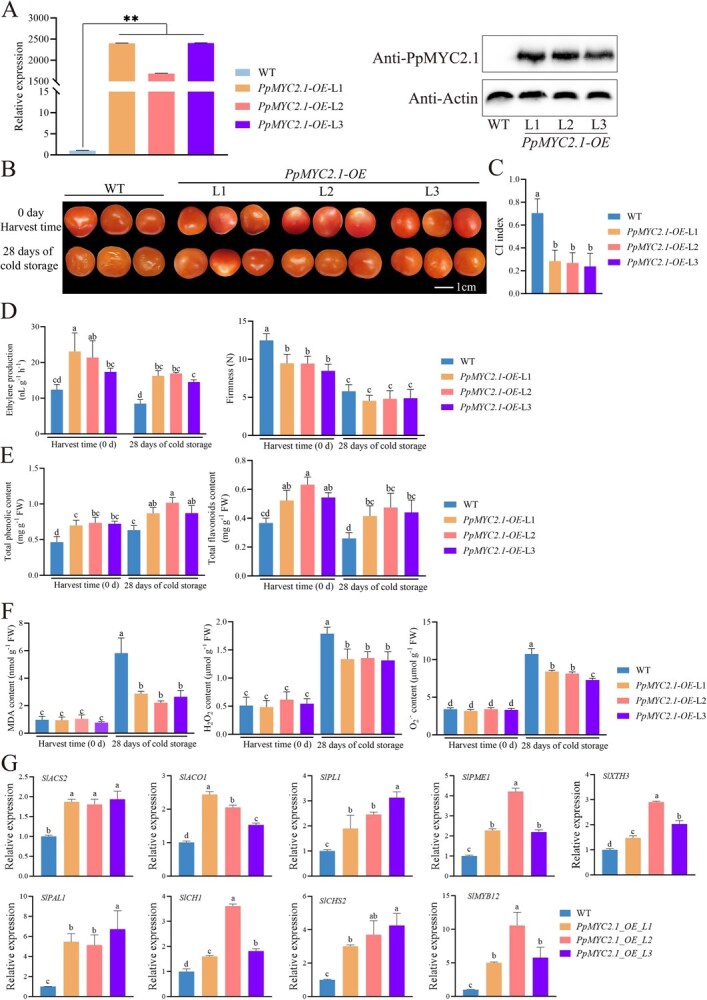
Overexpression of *PpMYC2.1* in transgenic tomato fruit enhances chilling tolerance. (A) RT-qPCR and western blot analyses confirmed stable overexpression of PpMYC2.1 in tomato fruits. (B, C) Phenotypic comparison and CI index of WT and *PpMYC2.1*-overexpressing tomato lines during cold storage. (D) Ethylene biosynthesis and fruit firmness of WT and *PpMYC2.1*-overexpressing lines during cold storage. (E) Total phenolic and flavonoids contents of WT and *PpMYC2.1*-overexpressing lines during cold storage. (F) MDA, O_2_^·−^ and H_2_O_2_ levels of WT and *PpMYC2.1*-overexpressing lines during cold storage. (G) Relative expressions of *SlACS2*, *SlACO1*, *SlPL1*, *SlPME1*, *SlXTH3*, *SlPAL1*, *SlCHI*, *SlCHS2,* and *SlMYB12* of WT and *PpMYC2.1*-overexpressing lines after 28 d of cold storage. Different letters indicate significant differences at *P* < 0.05 level (one-way ANOVA, Tukey's HSD).

To explore the functional role of PpMYC2.1 in regulating CI, we generated stable *PpMYC2.1*-overexpressing transgenic tomato plants. Three independent transgenic lines (L1, L2, and L3) were successfully obtained. RT-qPCR and immunoblot analyses confirmed robust accumulation of PpMYC2.1 transcripts and protein in the transgenic fruit, while no detectable expression was observed in wild-type (WT) fruit (Fig. 8A). Following 28 days of cold storage, transgenic tomato fruit displayed enhanced cold tolerance, exhibiting only mild CI symptoms with a CI index of approximately 0.3. In contrast, WT fruit developed severe CI symptoms, with a CI index of approximately 0.7 (Fig. 8B and C). Meanwhile, the transgenic lines exhibited significantly higher ethylene production and slightly reduced fruit firmness compared to WT fruit (Fig. 8D). Consistent with the observed CI phenotypes, the transgenic tomato lines showed reduced accumulation of MDA, H₂O₂, and O₂^·−^, alongside elevated levels of polyphenols and flavonoids (Fig. 8E and F). Moreover, the transcription of genes associated with ethylene biosynthesis (*SlACS2* and *SlACO1*), cell wall degradation (*SlPL1*, *SlPME1*, and *SlXTH3*), and the phenylpropanoid pathway (*SlPAL1*, *SlCHI*, *SlCHS1,* and *SlMYB12*) was upregulated in the transgenic lines (Fig. 8G). Notably, binding sites for PpMYC2.1 were identified in the promoter regions of these genes (Text S2), further supporting its direct regulatory role. Collectively, these results confirm that PpMYC2.1 contributes to enhanced cold tolerance in fruit by modulating ethylene production, antioxidant capacity, and cell wall remodeling.

## Discussion

### MYC2 as a conserved core regulator in JA-mediated cold tolerance across fruit species

MYC2 serves as a central regulatory node in JA signal transduction, orchestrating a wide range of physiological processes [38–40]. Accumulating evidence suggests that MYC2 is essential for mitigating CI in fruit. In banana, MaMYC2 interacts with MaICE1 to activate the ICE–CBF cold-responsive pathway under MeJA induction [21]. In tomato, SlMYC2 directly regulates polyamine biosynthesis by targeting *SlARG1*, *SlARG2*, *SlADC*, and *SlODC* [16]. Furthermore, SlMYC2 enhances the transcriptional activity of SlWRKY50, which in turn binds to the promoter of *SlAOS*, a key gene in JA biosynthesis, thereby significantly upregulating its expression and promoting endogenous JA synthesis [41]. In peach, two MYC2 homologs have been identified—PpMYC2.1 and PpMYC2.2*.* While *PpMYC2.2* shows negligible expression, *PpMYC2.1* is strongly induced by MeJA (Fig. 2B). Given its specific responsiveness, PpMYC2.1 is proposed as a key regulator of cold sensitivity in peach fruit. Elucidating its downstream network will not only advance our understanding of JA-mediated cold acclimation but also provide a prime target for breeding peach cultivars with enhanced cold resistance and maintained fruit quality.

### PpMYC2.1 directly regulates multiple genes to alleviate CI in peach fruit

CI in peach fruit is closely linked to alterations in hormone levels, lipid peroxidation, and imbalances in antioxidant metabolism [42, 43]. To elucidate the transcriptional regulatory mechanisms underlying PpMYC2.1-mediated cold tolerance, we integrated DAP-seq and RNA-seq analyses and identified 1920 putative target genes directly regulated by PpMYC2.1, including TFs involved in ethylene biosynthesis, as well as key genes related to cell wall degradation and phenylpropanoid metabolism (Fig. 3).

Ethylene, an essential plant hormone, regulates diverse physiological processes including fruit ripening, senescence, and stress responses [44]. Nonetheless, its influence on enhancing cold tolerance in peach fruit remains controversial, largely owing to cultivar-specific differences in genetic background and ethylene signaling pathways. This is particularly evident in SH peaches, which exhibit inherent ethylene biosynthesis deficiencies and consequently display heightened susceptibility to CI due to their impaired ability to activate ethylene-mediated stress responses. Recent studies have demonstrated that exogenous ethylene application can effectively alleviate CI symptoms in SH peaches by compensating for endogenous ethylene deficiency and restoring cold-responsive defense pathways [35]. However, whether direct exogenous ethylene treatment can effectively alleviate CI symptoms in ‘CP9’ remains to be further investigated. Supporting this ethylene-mediated CI reduction, earlier studies have also indicated that MeJA can stimulate ethylene production, thus decreasing CI in peach fruits [14, 15]; however, the molecular mechanisms driving this response have yet to be completely clarified. In apple, JA induces the expression of MdMYC2, which directly binds to the promoters of *MdACS1* and *MdACO1* to enhance their expression [23]. In contrast, our results indicate that PpMYC2.1 in peach does not directly activate the expression of *PpACS1* and *PpACO1* (Fig. S13, Data Set S1), but rather influences their expression by indirectly activating their upstream regulators PpIAA1 [45], PpHB.G7 [46], and PpERF61 [47] (Fig. 4). These findings suggest that PpMYC2.1 contributes to MeJA-induced ethylene biosynthesis in peach fruit by orchestrating a transcriptional regulatory cascade rather than directly activating ethylene biosynthesis genes.

Impaired fruit softening under low-temperature conditions is another hallmark symptom of CI, which compromises fruit textural quality and sensory acceptability while reducing market value [2, 43]. This phenomenon is primarily due to the downregulation of multiple genes responsible for cell wall degradation and modification, including *PpPG1*, *PpPG2*, *PpPG24*, *PpPL1*, *PpXTH2*, and *PpPME1*, which have been strongly associated with peach fruit softening [48–51]. Notably, MeJA has been shown to promote normal softening of fruit under low-temperature conditions, as observed in both peach [15] and mango [9]. However, the regulatory interaction between MYC2 and genes involved in cell wall modification in peach fruits is still not well understood. Here, we demonstrate that PpMYC2.1 directly interacts with the promoters of three genes responsible for encoding essential cell wall-modifying enzymes—*PpPL1*, *PpPG2*, and *PpXTH2*—thereby positively influencing their expression (Fig. 5). These findings reveal a direct regulatory role for PpMYC2.1 in the softening process of peach fruit, enhancing our understanding of the transcriptional network involved in MeJA-induced cell wall degradation under low-temperature conditions.

Phenolics and flavonoids act as potent antioxidants that scavenge ROS and suppress membrane lipid peroxidation [52]. These metabolites are produced via an intricate sequence of enzymatic reactions that occur within the phenylpropanoid metabolic pathway [53]. MeJA treatment has been shown to activate this pathway and enhance the biosynthesis of related secondary metabolites, thereby improving cold tolerance in several fruits, including pear [54], *Rosa roxburghii* [18], and litchi [55], but the molecular mechanisms bridging MeJA signaling to phenylpropanoid metabolism in peach remain poorly defined. In tomato, the SlMYC2–MED25 complex promotes flavonoid accumulation by directly binding to the promoter of *SlMYB12*, which upregulates *FLS*, *F3*′*H*, *F3H*, and *CHS* [22]. In contrast to this indirect regulatory mechanism, our present study demonstrates that PpMYC2.1 directly influences the expression of key structural genes within the phenylpropanoid metabolic pathway, including *PpPAL1*, *Pp4CL*, *PpCHI3*, and *PpCHS*, thereby exerting a direct influence on polyphenol and flavonoid biosynthesis (Fig. 6). Given the absence of a stable genetic transformation system in peach, which impedes the faithful reconstruction of endogenous regulatory networks, we employed transient overexpression or silencing of *PpMYC2.1* in peach fruit flesh and stable overexpression of *PpMYC2.1* in tomato as alternative approaches to validate its function (Fig. 8). Since all approaches consistently enhanced chilling tolerance, we conclude that PpMYC2.1 functions as a key transcriptional hub that coordinates ethylene biosynthesis, cell wall remodeling, and phenylpropanoid metabolism to mitigate CI, thereby maintaining antioxidant capacity and hormonal balance in peach fruit under cold stress.

### PpJAZ2/4-PpMYC2.1 module is involved in MeJA-mediated alleviation of CI in peach fruit

In plants, JAZ proteins serve as crucial inhibitors of JA signaling by suppressing the activity of various TFs, including members of the WRKY, MYC2/3/4/5, MYB, and MADS families [56–58]. Among these, JAZ proteins are particularly well known for modulating MYC-dependent transcriptional cascades that orchestrate diverse defense responses. In rice, for example, OsJAZ11 represses OsMYC2 activity, thereby modulating the expression of key genes, *OsMADS29* and *OsMADS68*, and negatively regulating spikelet development [59]. In tomato, SlJAZ4 interacts with SlMYC2 and negatively regulates resistance to *Helicoverpa armigera* [32]. Despite these advances, the regulatory function of the JAZ–MYC2 transcriptional module in peach remains unexplored. In the present study, we examined the transcriptional profiles of JAZ genes in MeJA-treated peach fruit under low-temperature conditions and found that *PpJAZ2* and *PpJAZ4* were markedly downregulated. Interaction assays confirmed that both bind to PpMYC2.1 and inhibit its transcriptional activity (Fig. 7), consistent with known JAZ–MYC2 repression mechanisms.

JAZ proteins are typically degraded via the COI1-mediated ubiquitin-proteasome pathway [60], with degron sequences playing critical roles in COI1 recognition [25, 61]. We found that MeJA treatment significantly suppressed *PpCOI1* expression (Fig. S2F). This suppression likely contributed to the upregulation of *PpJAZ1, PpJAZ3, PpJAZ5,* and *PpJAZ10* expression*,* all of which harbor canonical degron sequences (Fig. S14, Text S1). PpJAZ7, which contains an atypical degron sequence (AGMSMK) not previously reported, which may still retained its ability to bind PpCOI1 (Fig. S14). PpJAZ2 contains an FPIARR degron similar to the canonical LPIAR(R/K) motif; however, this variant has been shown to lack COI1-binding ability in strawberry [61]. Although PpJAZ4 harbors canonical degron sequences, its expression was nonetheless inhibited, suggesting the involvement of alternative regulatory mechanisms. Recent studies have shown that post-translational modifications, such as MPK6 or SnRK1.1-mediated phosphorylation and PUB22/SINA11-dependent ubiquitination, also contribute to JAZ protein degradation [32, 61, 62]. Our data provide initial evidence that PpJAZ2 and PpJAZ4 are candidate substrates for phosphorylation or ubiquitination (Tables S4 and S5). Furthermore, MeJA treatment significantly induced the expression of the E3 ubiquitin ligase *PpPUB23* and the kinase *PpSnRK1* (Fig. S15), suggesting their potential role in regulating PpJAZ2/4 degradation and highlighting the need to investigate their specific functions in JA signaling.

**Figure 9:**
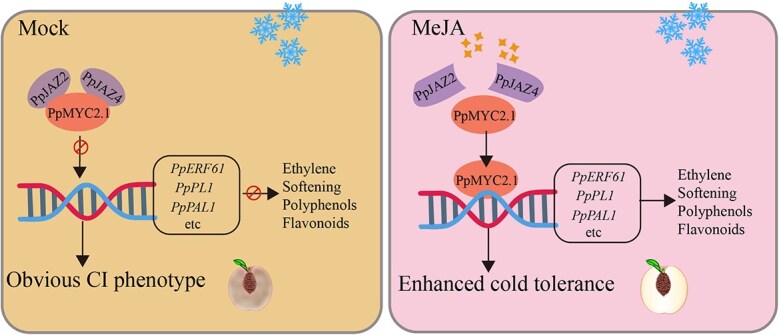
Proposed working model demonstrating the positive regulatory function of PpMYC2.1 in improving cold tolerance. Under cold stress conditions, PpJAZ2 and PpJAZ4 physically interact with PpMYC2.1, suppressing its transcriptional activation of downstream genes and thereby inhibiting JA signaling. This repression leads to reduced expression of downstream genes and exacerbates CI symptoms in peach fruit. However, exogenous MeJA treatment downregulates the expression of *PpJAZ2* and *PpJAZ4*, thereby alleviating their inhibitory effects on PpMYC2.1. As a result, JA signaling triggers the expression of downstream genes involved in ethylene biosynthesis, fruit softening, and polyphenol/flavonoid synthesis, ultimately enhancing the cold tolerance of peach fruit.

Based on the aforementioned research findings, we present a conceptual model illustrating the positive regulatory role of PpMYC2.1 in alleviating CI in peach fruit (Fig. 9). Under cold stress conditions, PpJAZ2 and PpJAZ4 interact with PpMYC2.1, suppressing its transcriptional activation activity and thereby attenuating JA signaling, resulting in evident CI symptoms in peach fruit. However, exogenous MeJA treatment downregulates the expression of *PpJAZ2* and *PpJAZ4*, thereby attenuating their inhibitory effect on PpMYC2.1. As a result, both transcriptional and protein levels of PpMYC2.1 increase, reactivating JA signaling. This reactivation, in turn, induces the expression of downstream genes linked to ethylene biosynthesis, fruit softening, and polyphenol/flavonoid biosynthesis, ultimately enhancing cold tolerance in peach fruit.

## Materials and methods

### Plant material and MeJA treatments

SH peach fruit (*Prunus persica* L. Batsch cv. Chinese peach 9/‘CP 9’) were used as the experimental material in this study. Fruits were collected at the stage of commercial maturity (95 days after full bloom) from an orchard located in Xinxiang, Henan Province, China. Uniform fruits—similar in size, free from defects, pests, and mechanical injuries—were selected for experimentation. Approximately 600 fruits were randomly assigned to two treatment groups, each consisting of approximately 300 peaches. In the MeJA treatment group, peaches were submerged in a 1 mM MeJA solution with 0.1% Triton X-100 for 15 minutes, while those in the control group were treated with a solution containing only 0.1% Triton X-100. Following treatment, fruits were stored at 4°C with 90% relative humidity for up to 28 days. Sampling was performed at 7-day intervals, with three biological replicates collected per treatment at each time point, each replicate consisting of six fruits. Flesh tissues were excised, rapidly frozen in liquid nitrogen, and subsequently stored at −80°C for future analysis.

### Quality assessment

The evaluation of fruit firmness, ethylene production, and IB was conducted following previous studies performed in our laboratory [63]. To assess firmness, a 1 mm thick slice of peel was excised from the equatorial region of each fruit. Firmness measurements were taken on opposite sides of the fruit utilizing a TA.XTC20 texture analyzer (BosinTech, Shanghai, China) fitted with a cylindrical probe of 7.5 mm in diameter.

Ethylene production was measured by placing two peach fruits in a sealed 1 l container for 2 hours. Subsequently, a 1 ml sample of the headspace gas was collected and analyzed using a gas chromatograph GC-2010 (Shimadzu, Kyoto, Japan).

CI in SH peach fruit was primarily assessed based on the severity of IB, a hallmark symptom of cold damage. The IB index was calculated using the following formula: IB index = 100% × Σ [(internal browning level) × (number of fruits at that level)]/[4 × total number of fruits assessed]. Fruits were classified into five levels based on the extent of browning: Level 0 indicates no visible browning in the flesh (0% of tissue affected); Level 1 indicates 1% to 25% of tissue browned; Level 2 indicates 26–50% of tissue browned; Level 3 indicates 51% to 70% of tissue browned; and Level 4 indicates 76% to 100% of tissue browned.

The CI index, used to assess CI severity in tomato fruits during cold storage, was calculated via visual recording of symptoms based on the following criteria: Level 0 (no symptoms), Level 1 (<5% surface area affected), Level 2 (5%–25%), Level 3 (25%–50%), and Level 4 (>50%). The CI index = Σ [(CI level) × (number of fruits at that level)]/[4 × total number of fruits assessed].

For biochemical analyses, the contents of MDA, H₂O₂, and O_2_^·−^, along with enzymatic activities of PPO and POD in peach fruit, were determined using commercial assay kits (Solarbio, Beijing, China). Briefly, 0.1 g of the sample was homogenized and prepared following the manufacturer’s instructions. Absorbance readings were taken at specific wavelengths by Microplate Photometer (Thermo Fisher Scientific, New York, NY, USA), and the results were expressed as follows: MDA, nmol g^−1^ FW; H₂O₂, μmol g^−1^ FW; O_2_^·−^, nmol g^−1^ FW; and PPO and POD activities, U mg^−1^ prot. The contents of JA and JA-Ile were determined by ELISA kits (Meimian, Jiangsu, China).

### RNA-seq and data analysis

The RNA-seq project was conducted by Novogene Co., Ltd. (Beijing, China). Raw sequencing reads were processed with Trimmomatic v0.36 [64]. Clean reads were then aligned to the *P. persica* reference genome v2.0 (available at http://www.peachmd.com/#/download) using HISAT2 v2.2.2 [65]. Gene expression levels were quantified using Subread v2.0.7 [66]. DEGs were identified using DESeq2 v1.32.0 [67], with significance thresholds set at |log2(FoldChange)| ≥ 1 and adjusted *P* value (*P*_adj_) ≤ 0.05. Pathway enrichment analysis of DEGs was performed using Peachmd (http://www.peachmd.com/#/enrichment).

### DAP-seq and data analysis

DAP-seq was conducted by Bluescape Scientific Co., Ltd (Hebei, China). A recombinant PpMYC2.1 protein fused with a HaloTag sequence was synthesized using the ProteoLiposome PLUS Expression Kit (TIANGEN, Beijing, China). The DAP-seq DNA library was constructed using the TLX DNA-Seq Kit (MICH, Baoding, China). The fusion protein (PpMYC2.1-HaloTag) or HaloTag protein alone (used as a negative control) was incubated with the DNA library. After binding, DNA fragments associated with the fusion protein were eluted from the beads. Two technical replicates and an input control were prepared for each sample. High-throughput sequencing was conducted on the DNBSEQ-T7 platform (MGI, Shenzhen, China), producing 150 bp paired-end reads for downstream analysis.

Raw sequencing reads were quality-filtered using fastp v0.23.4 [68]. Clean reads were aligned to the *P. persica* genome utilizing BWA-MEM v2.2 [69]. Peak calling was conducted using MACS2 v2.2.8, applying a *q*-value threshold of <0.05 to determine significant DAP-seq binding regions [70]. To identify enriched DNA-binding motifs in peak regions, MEME-chip v5.5.7 was employed [71]. Peak annotations were conducted with ChIPseeker v1.42.1 [72] to determine their relative positions to genes, and the distribution of DAP-seq read density around TSS was analyzed using deepTools2 v3.5.5 [73]. The enriched binding peaks were ultimately visualized using IGV v2.18.1 [74].

### Subcellular localization analysis

The full-length coding sequences of *PpMYC2.1*, *PpJAZ2*, and *PpJAZ4* were cloned into the pSuper1300-GFP vector to generate GFP fusion constructs (*PpMYC2.1*-GFP, *PpJAZ2*-GFP, and *PpJAZ4-GFP*, respectively). The resulting recombinant plasmid was transiently expressed in *N. benthamiana* leaves via *Agrobacterium*-mediated infiltration. GFP and mCherry fluorescence signals of the fusion protein were imaged using a fluorescence microscope (Leica Microsystems, Wetzlar, Germany).

### DLR assay

The promoter regions of the target genes were individually cloned into the pGreenII 0800-LUC as reporter, while the full-length coding sequence of *PpMYC2.1* was cloned into the pGreenII 62-SK vector as effector. All constructs were transformed into *Agrobacterium tumefaciens* GV3101 (pSoup), and co-infiltrated into *N. benthamiana* leaves at a 9:1 (reporter/effector) ratio. After 48 hours of incubation, absolute LUC and REN activities were measured using a dual-luciferase assay kit (YEASEN, Shanghai, China). Transcriptional activation of target promoters was assessed by calculating the LUC/REN ratio. The SK empty vector co-infiltrated with each promoter construct served as a negative control. Each luciferase activity was tested in at least six independent replicates.

### EMSA

The coding sequence of *PpMYC2.1* was inserted into the pET-SUMO vector to produce a His-tagged fusion protein. The PpMYC2.1-His construct and the empty pET-SUMO vector (a negative control) were transformed into *Escherichia coli* strain BL21(DE3) and induced with isopropyl β-d-thiogalactoside at 16°C. The recombinant protein was purified using Anti-His Magnetic Beads (Beyotime, Shanghai, China). Based on DAP-seq results, promoter fragments were synthesized (TSINGKE, Beijing, China) and labeled using the Biotin 3′ End DNA Labeling Kit (Beyotime). EMSA was performed using the Chemiluminescent EMSA Kit (Beyotime).

### Y1H assay

The coding sequence of *PpMYC2.1* was inserted into the pGADT7 vector, resulting in the construction of the pGADT7-PpMYC2.1. Promoter sequences (200–400 bp) of the target genes were ligated into the pAbAi vector. The resulting pAbAi constructs were transformed into Y1HGold yeast cells and cultured on SD/-Ura medium (Coolaber, Beijing, China) for selection. Positive colonies were transferred to SD/-Ura solid medium supplemented with varying concentrations of AbA to determine the minimal AbA concentration required to suppress self-activation. Subsequently, the pGADT7-PpMYC2.1 plasmid was introduced into the positive colonies and selected on SD/-Leu medium (Coolaber) containing the optimized AbA concentration for further interaction screening.

### Gene expression and immunoblot analysis

Total RNA was extracted from peach and tomato fruit using the EASYspin Plus Plant RNA kit (Aidlab Biotech, Beijing, China). First-strand cDNA was synthesized using HiScript III All-in-one RT SuperMix (Vazyme, Nanjing, China). RT-qPCR was performed with Taq Pro Universal SYBR qPCR Master Mix (Vazyme) on a Roche LightCycler® 480 (Roche, Basel, Switzerland).

The polyclonal antibodies against PpMYC2.1, PpJAZ2, and PpJAZ4 were affinity-purified from rabbit antisera by Shanghai Abmart Biotechnology Co., Ltd (Shanghai, China). Total protein was extracted from peach and tomato fruit using Plant Cell Lysis Buffer (Beyotime). Equal amounts of protein samples were separated using a 12.5% (w/v) SDS-PAGE gel and subsequently electrotransferred onto a 0.45-μm PVDF membrane (Epizyme, Shanghai, China) using a Bio-Rad transfer system (Bio-Rad, USA). Immunoblotting analysis was performed using the respective primary antibodies, accompanied by a secondary goat anti-rabbit IgG peroxidase antibody (Epizyme).

### Y2H, LCI, BiFC and Co-IP assays

For the Y2H assay, the coding sequence of *PpMYC2.1* was cloned into the pGBKT7 vector to generate binding domain (BD)-PpMYC2.1, while the coding sequences of *PpJAZ2* and *PpJAZ4* were inserted into the pGADT7 vector to construct activation domain (AD)-PpJAZ2 and AD-PpJAZ4, respectively. The resulting BD-PpMYC2.1 and AD-PpJAZ2/4 constructs were co-transformed into the Y2HGold yeast strain and plated onto SD/-Leu/-Trp or SD/-Leu/-Trp/-His/-Ade. Transformants containing empty vectors (BD or AD) were utilized as negative controls.

For the LCI assay, the coding sequence of *PpMYC2.1* was cloned into the pCAMBIA1300-NLUC vector to generate PpMYC2.1-NLUC, while the coding sequences of *PpJAZ2* and *PpJAZ4* were cloned into the pCAMBIA1300-CLUC vector to produce CLUC-PpJAZ2 and CLUC-PpJAZ4, respectively. The resulting constructs were introduced into *N. benthamiana* leaves as described above. Luciferase activity was detected 48 hours after infiltration by spraying the leaves with 1 mM D-luciferin, incubating for 5 minutes in the dark, and capturing the signal using a chemiluminescence imaging system (BIO-OI, Guangzhou, China).

For the BiFC assay, the coding sequence of *PpMYC2.1* and PpJAZ2/4 were cloned into the pSPYNE and pSPYCE vectors, respectively. The resulting vectors, along with empty plasmids as control, were transiently into *N. benthamiana* leaves as described above. YFP fluorescence was detected 48 hours post-infiltration.

For the Co-IP assay, the coding sequence of *PpMYC2.1* was cloned into the pSuper1300-Flag vector to generate *PpMYC2.1*-Flag. Constructs for *PpJAZ2/4*-GFP and *PpMYC2.1*-Flag were transiently expressed in *N. benthamiana* leaves. After 48 hours of incubation, total proteins were extracted from the leaves as described above and immunoprecipitated with anti-GFP magnetic beads (Epizyme) at 4°C overnight to isolate the protein that has been tagged with an epitope. The eluted proteins were examined through immunoblotting using anti-GFP (Epizyme) and anti-Flag (Epizyme) antibodies, respectively, to detect potential protein–protein interactions.

### Plant transformation

To generate *PpMYC2.1*-overexpressing tomato lines, the full-length coding sequence was cloned into the PCAMBIA1301-GFP-flag vector. The resulting constructs were transformed into tomato plants via *A. tumefaciens* strain GV3101. Transgenic plants were selected based on antibiotic resistance and verified by RT-qPCR and western blot analysis. Three independent T2 transgenic lines were used for subsequent experiments. To assess cold susceptibility, mature red-stage fruits from WT and transgenic lines were stored at 4°C for 28 days.

Transient overexpression and silencing in peach fruits were conducted following the protocol of Wei *et al.* [75]. For constructing transient virus-induced gene silencing vectors, the *PpMYC2.1* fragment was amplified and inserted into the pTRV2 vector, generating pTRV2-*PpMYC2.1*. The peach fruits were immersed in a 5% NaClO solution for surface sterilization for 20 minutes. The surface moisture of the fruits was dried with sterile filter paper. A 1-cm-thick flesh slice was cut along both sides of the fruit suture line and inoculated onto MS medium for pre-culturing for 24 hours. For overexpression, *Agrobacterium tumefaciens* strains carrying the *PpMYC2.1*-GFP construct and an empty construct (as a negative control) were then infiltrated, respectively. For silencing, *Agrobacterium tumefaciens* strains carrying the pTRV2-*PpMYC2.1* and pTRV1 were mixed at a 1:1 ratio, and then used for infiltrated. After vacuum infiltration, flesh cubes were co-cultured on MS medium at 4°C for 14 days. Finally, the samples were flash-frozen in liquid nitrogen and stored at −80°C for subsequent use.

### Statistical analysis

All experiments were performed with at least three biological replicates to ensure data reliability. For comparisons between two groups, the unpaired Student’s *t*-test was used (^*^P < 0.05, ^**^P < 0.01). For comparisons involving multiple groups, a one-way analysis of variance (ANOVA) was performed, followed by Tukey’s honestly significant difference (HSD) test.

### Accession numbers, primers, and probe sequences

Accession numbers for all relevant sequences are listed in Table S3. Detailed information on vectors, restriction enzyme sites, as well as primer and probe sequences used for PCR and EMSA experiments, is provided in Table S6.

## Ethics declarations

Ethics approval and consent to participate.

## Supplementary Material

Web_Material_uhaf295

## Data Availability

The transcriptome data underlying this article are available in the China National Center for Bioinformation (CNCB) repository (https://ngdc.cncb.ac.cn/bioproject/) under the BioProject ID: PRJCA042417.
