# Supplementary material for: JAZ2/JAZ4-MYC2.1 module mediates MeJA-induced alleviation of chilling injury in peach fruit (Prunus persica)
Source: Hortic Res. 2025 Nov 3;13(2):uhaf295. doi: 10.1093/hr/uhaf295 (PMC12923268; doi:10.1093/hr/uhaf295)
Supplement: Web_Material_uhaf295 [file web_material_uhaf295.zip › Supplemental figures.docx]

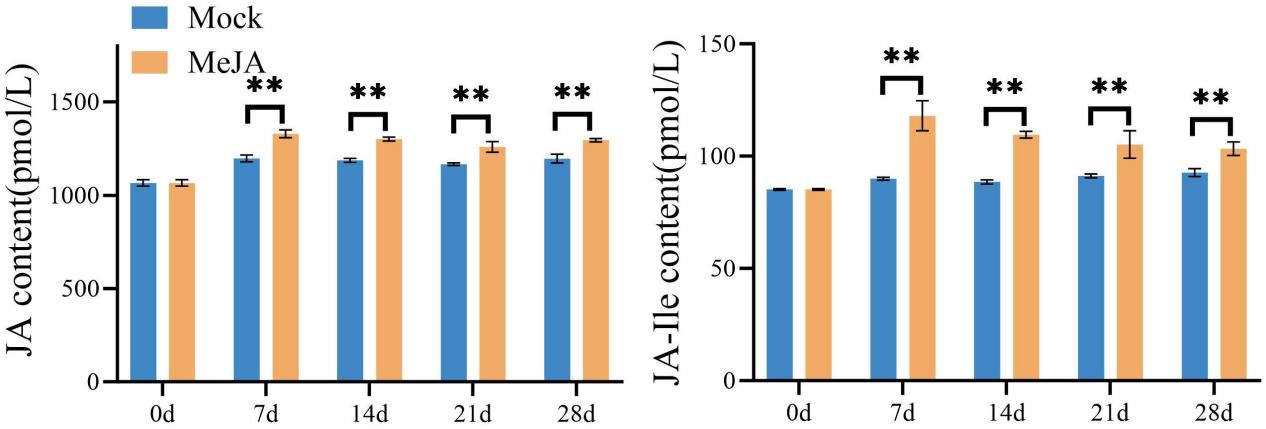


Figure S1. Impact of MeJA application on JA and JA-Ile contents in peach fruit during cold storage. Error bars represent means ± SE (n = 3 biological replicates). Asterisks indicate significant differences as determined by Student's *t*-test, with *P < 0.05 and **P < 0.01.


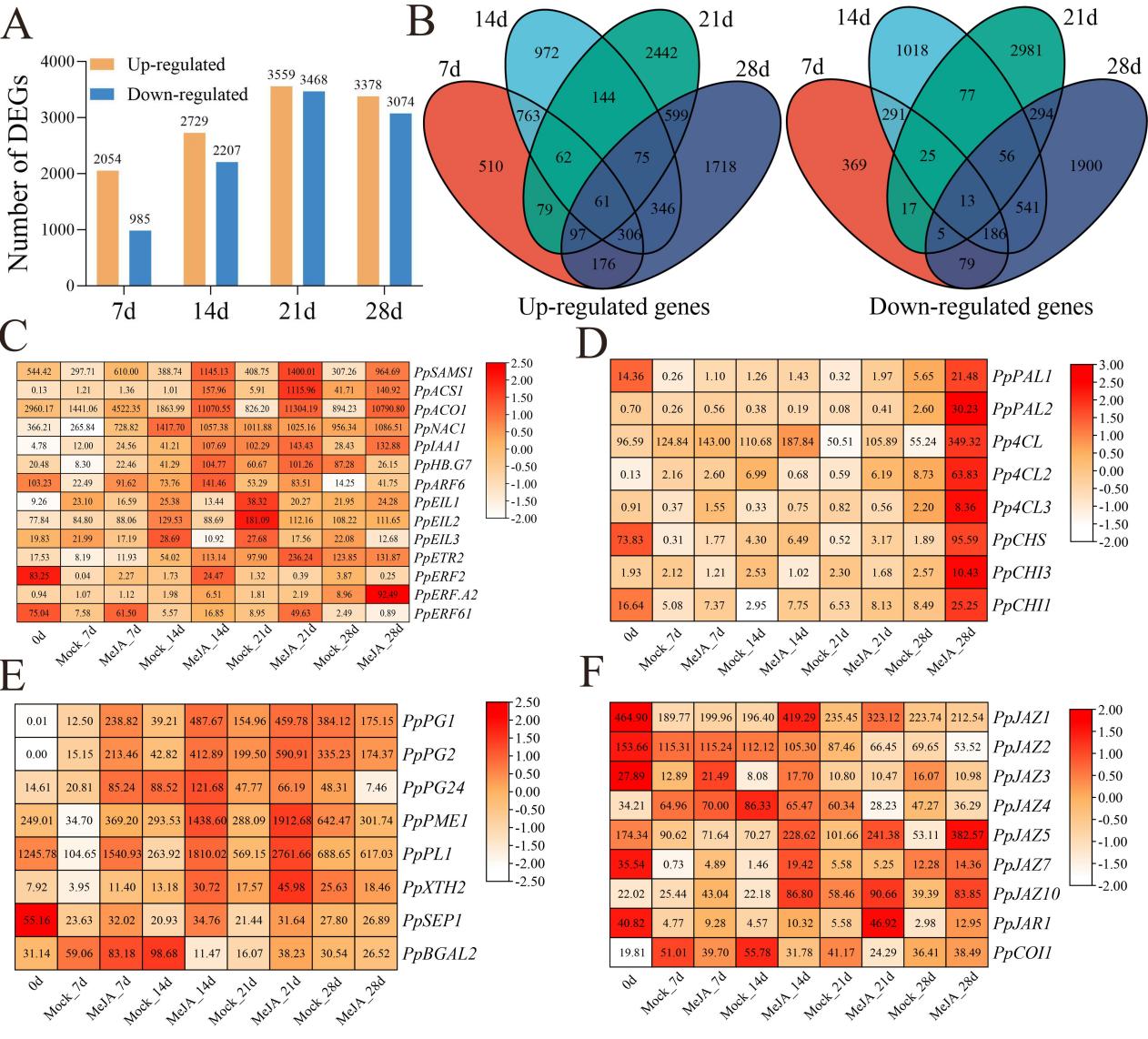


Figure S2. Impact of MeJA application on gene expression in peach fruit during cold storage.

**(A)** Number of differentially expressed genes (DEGs) identified between mock-treated and MeJA-treated fruit at 7, 14, 21, and 28 d, respectively. **(B)** Comparison of the overlapping number of DEGs during cold storage in MeJA treatment. **(C-F)** Heatmaps illustrating the expression profiles of DEGs involved in ethylene biosynthesis **C)**, phenolic metabolism **D)**, cell-wall degradation **E)**, and JA signaling pathway **F)**.


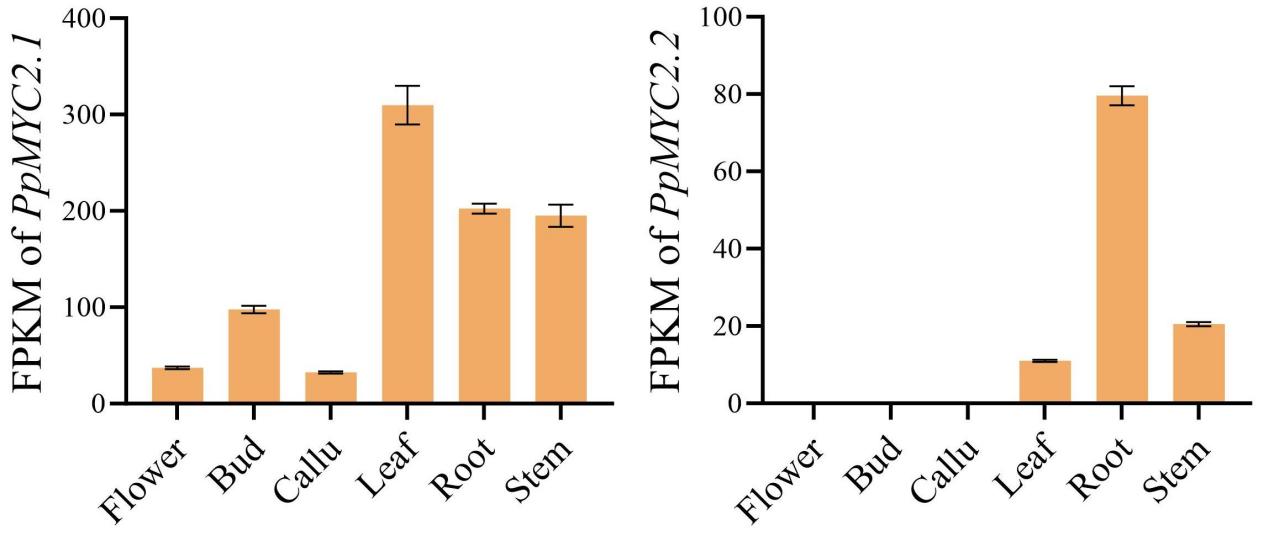


Figure S3. Expression patterns of *PpMYC2.1* and *PpMYC2.2* across different tissues. Data sourced from Peachmd (http://www.peachmd.com/#/trans). Error bars represent means ± SE (n = 3 biological replicates).


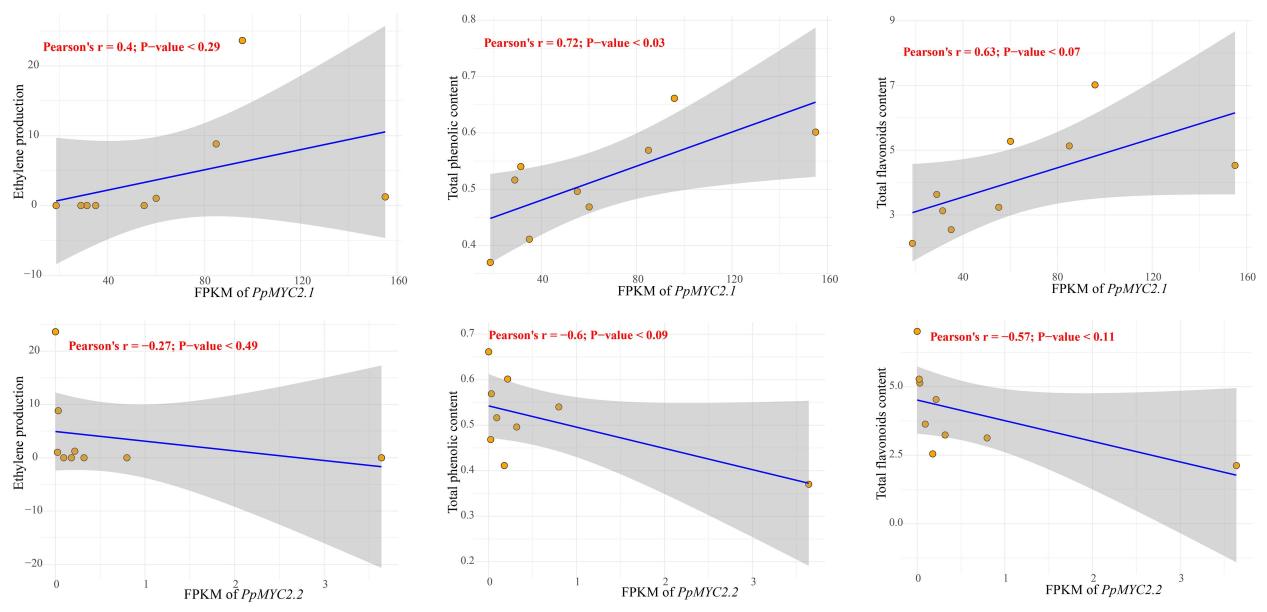


Figure S4. Correlation analysis between the expression of *PpMYC2.1*/*PpMYC2.2* and ethylene biosynthesis and polyphenol and flavonoid accumulation.


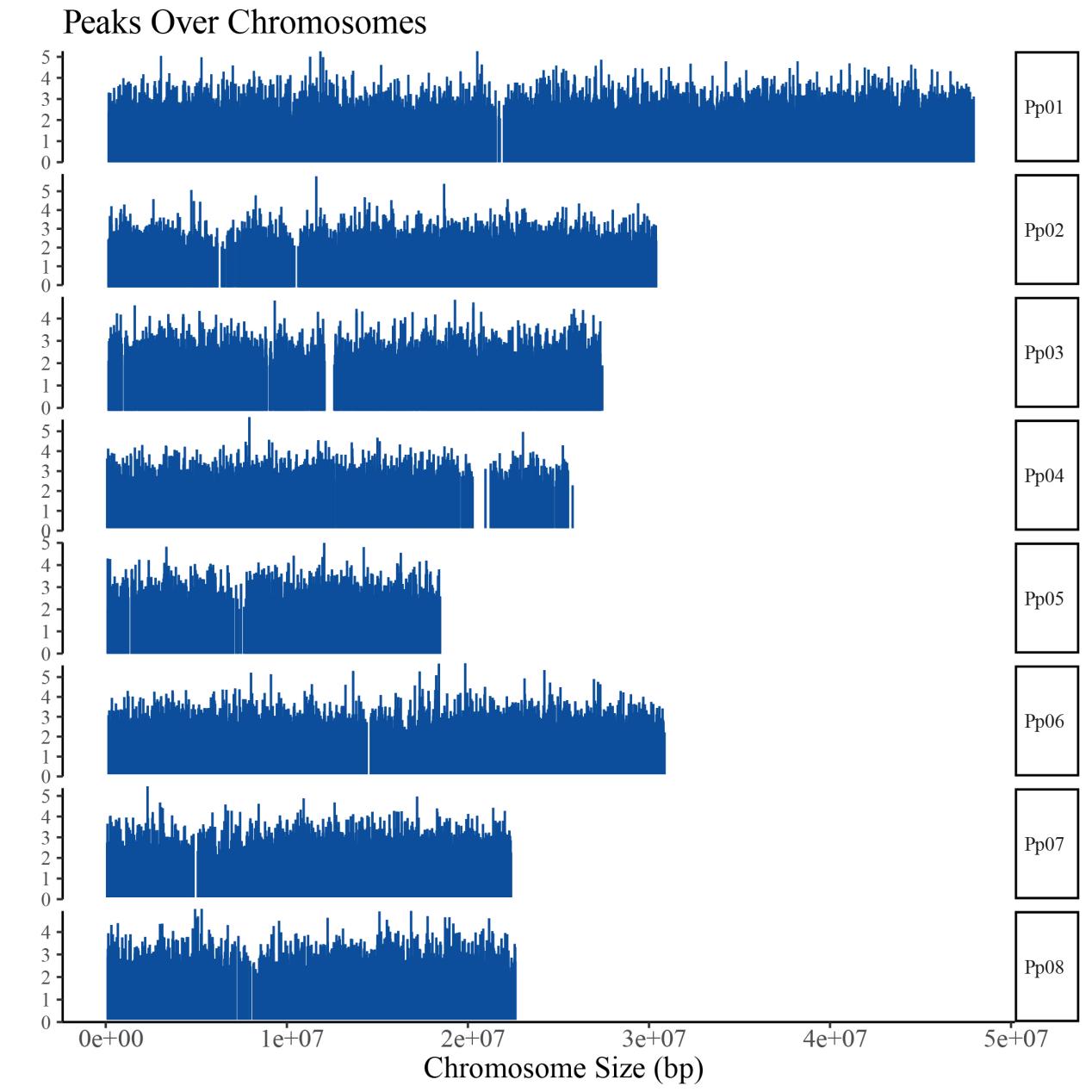


Figure S5. Genome-wide distribution of PpMYC2.1-binding sites across the eight *Prunus persica* chromosomes.


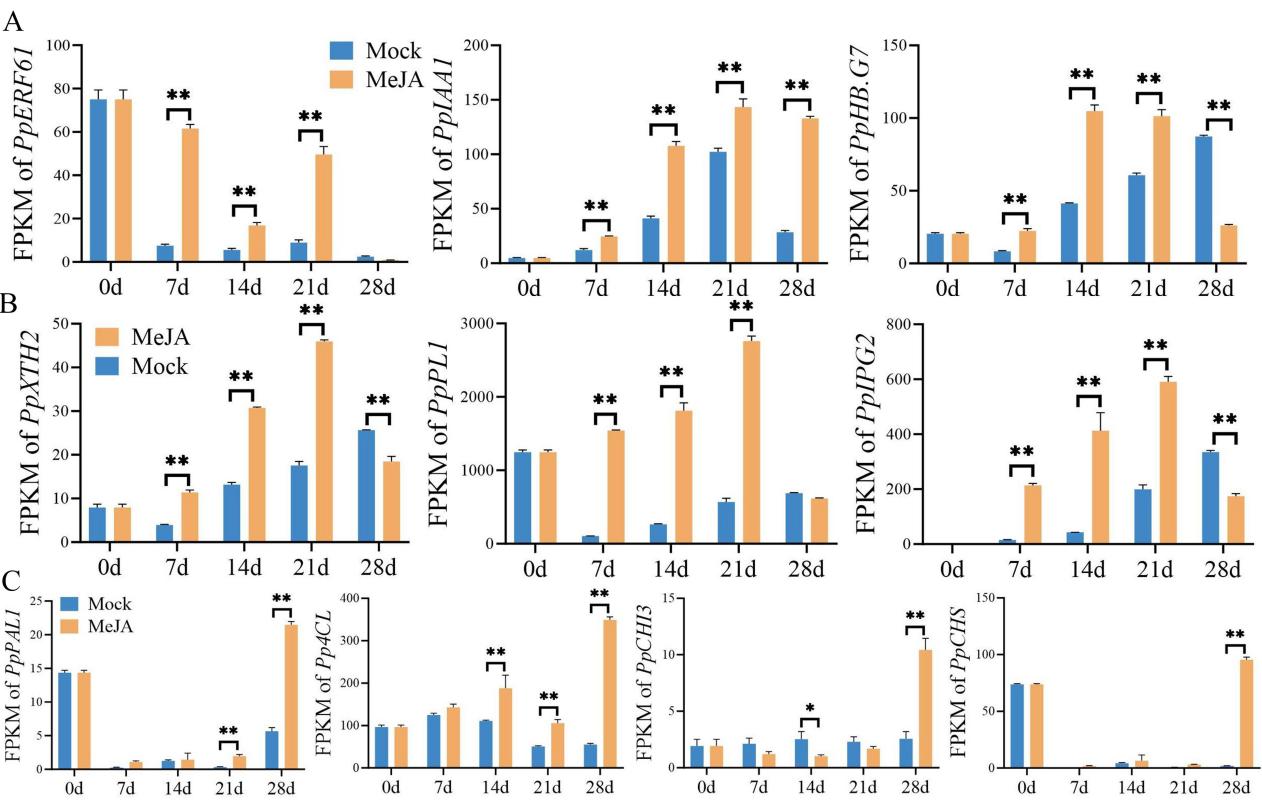


Figure S6. Expression levels of direct PpMYC2.1 target genes. Expression of genes associated with ethylene biosynthesis (A), cell-wall degradation (B), and polyphenol synthesis (C). Error bars represent means ± SE (n = 3 biological replicates). Asterisks indicate significant differences as determined by Student's *t*-test, with *P < 0.05 and **P < 0.01.


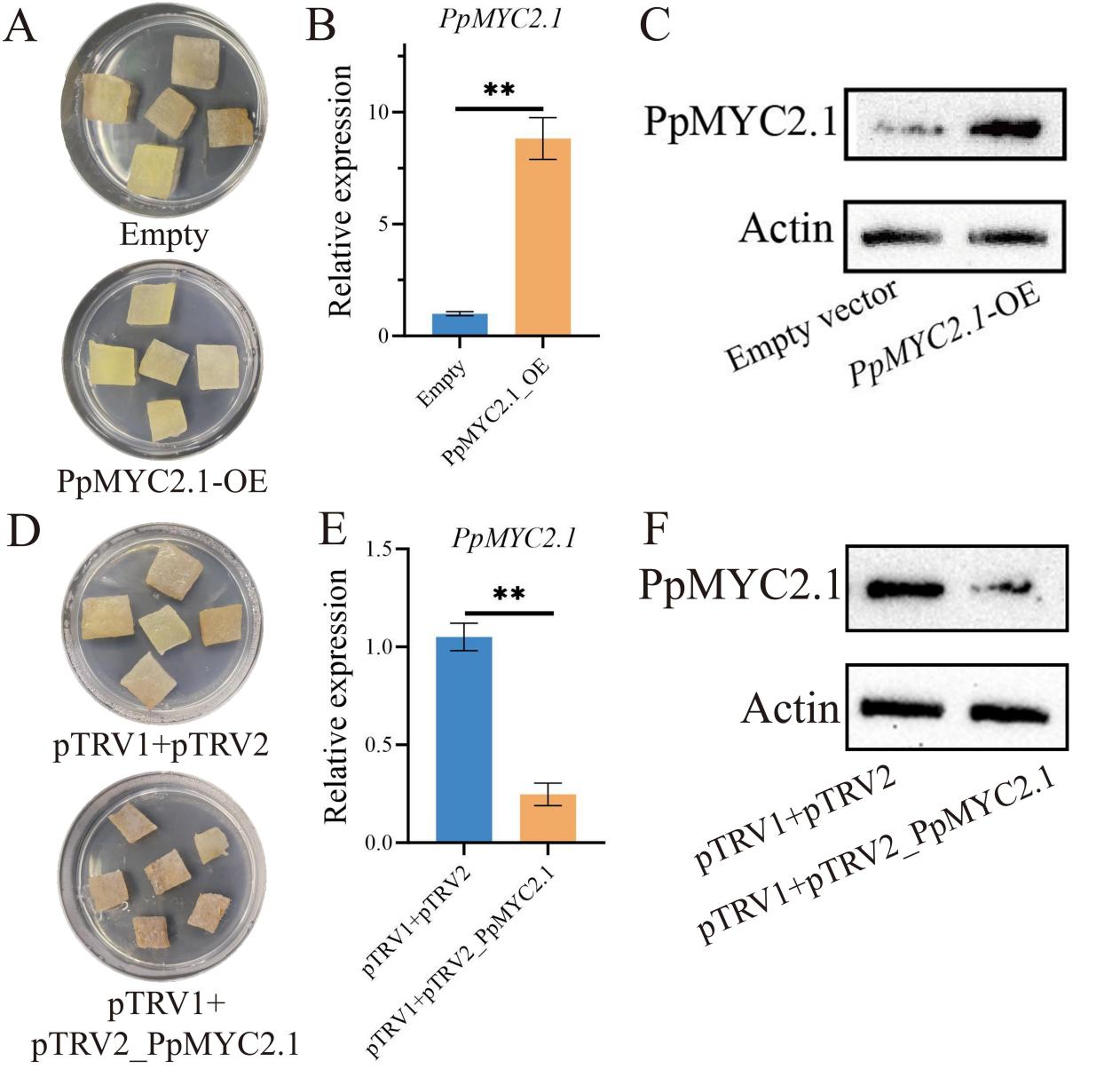


Figure S7. RT-qPCR and western blot analyses of PpMYC2.1 in the infiltrated peach fruit.

(**A**) Photograph of peach fruit flesh after transient overexpression of the control (empty vector) and PpMYC2.1-OE. (**B, C**) Gene expression **B**) and PpMYC2.1 protein accumulation **C**) in the flesh tissues infiltrated with PpMYC2.1 or empty vector (control). (**D**) Photograph of peach fruit flesh after virus-induced gene silencing with both pTRV1 and either pTRV2 (control) or pTRV2-PpWRKY14. (**E, F**) Gene expression **E**) and PpMYC2.1 protein accumulation **F**) with both pTRV1 and either pTRV2 or pTRV2-PpMYC2.1. Error bars represent means ± SE (n = 3 biological replicates). Asterisks indicate significant differences as determined by Student's *t*-test, with *P < 0.05 and **P < 0.01.

.

.


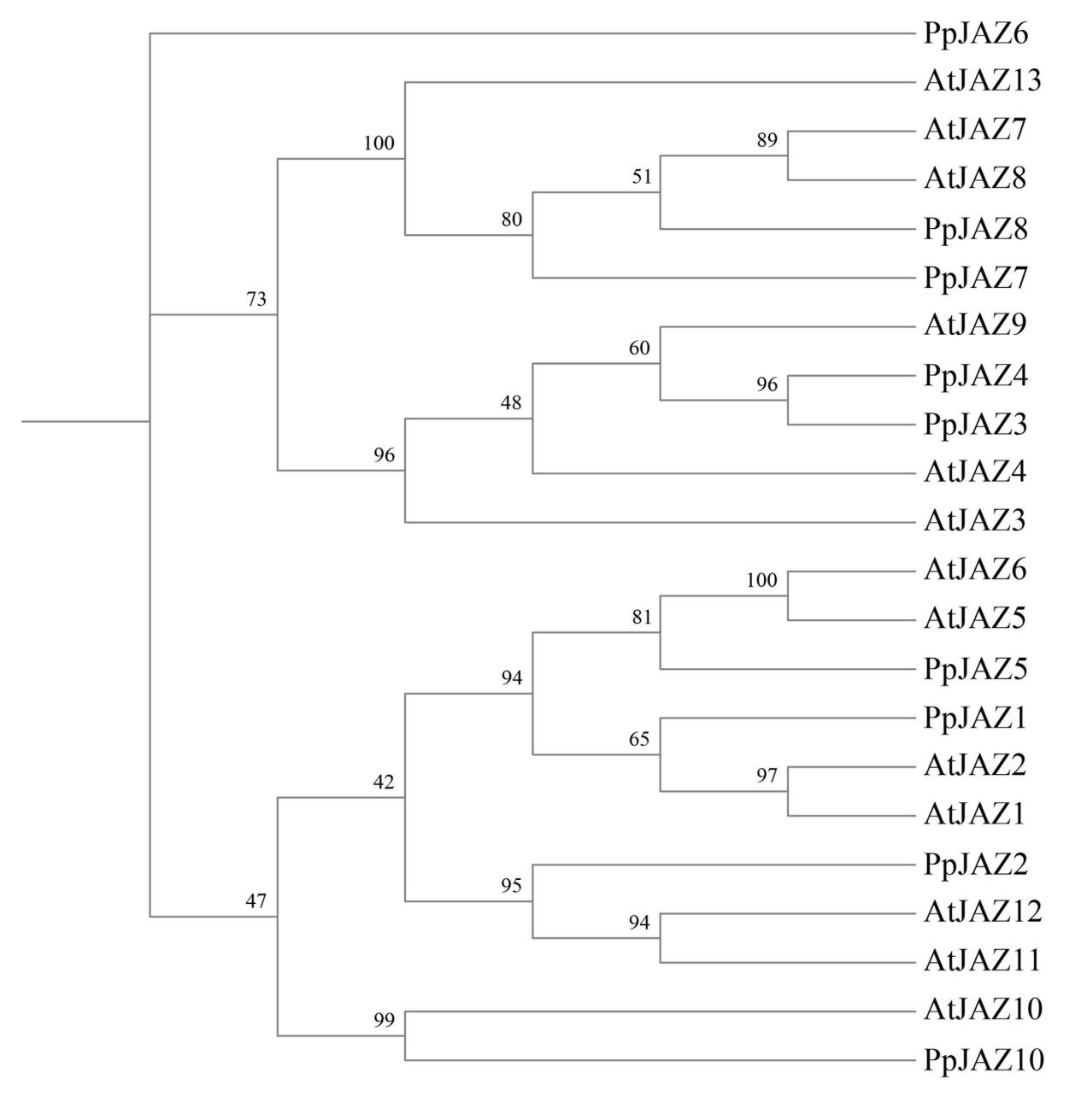


Figure S8. Phylogenetic tree of JAZ family members. At, *Arabidopsis thaliana*; Pp, *Prunus persica.*


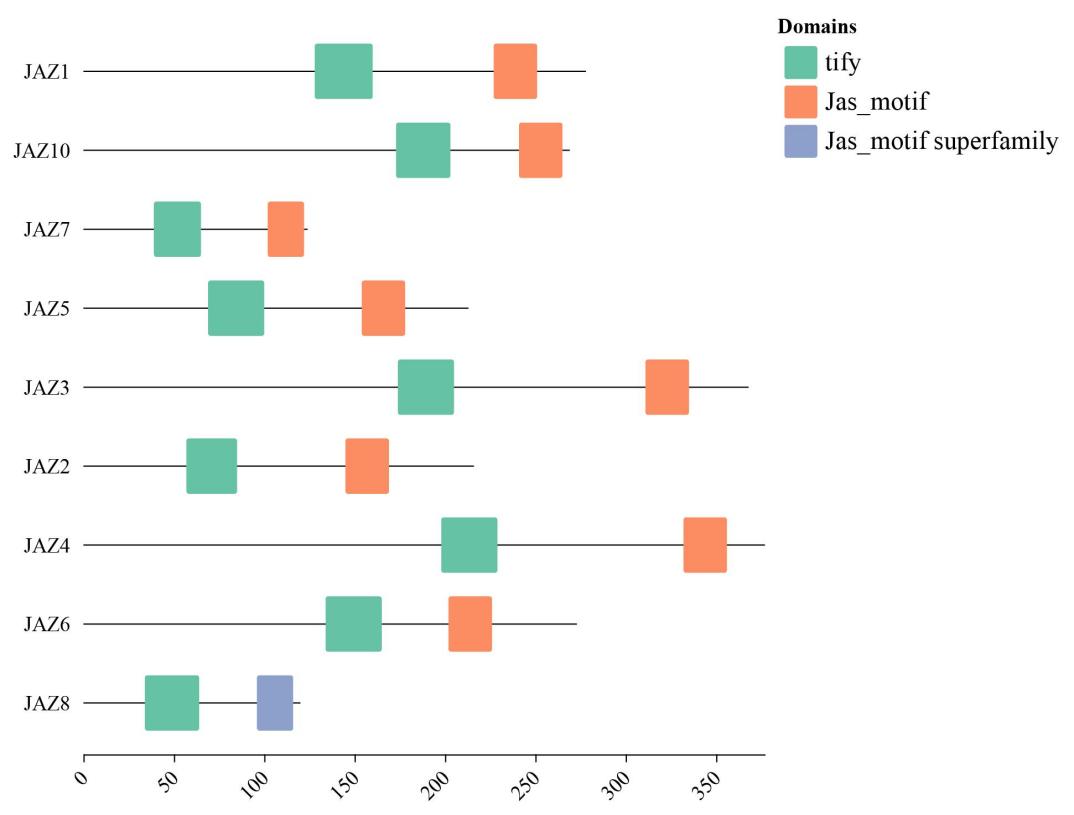


Figure S9. Conserved domain analysis of PpJAZ proteins.


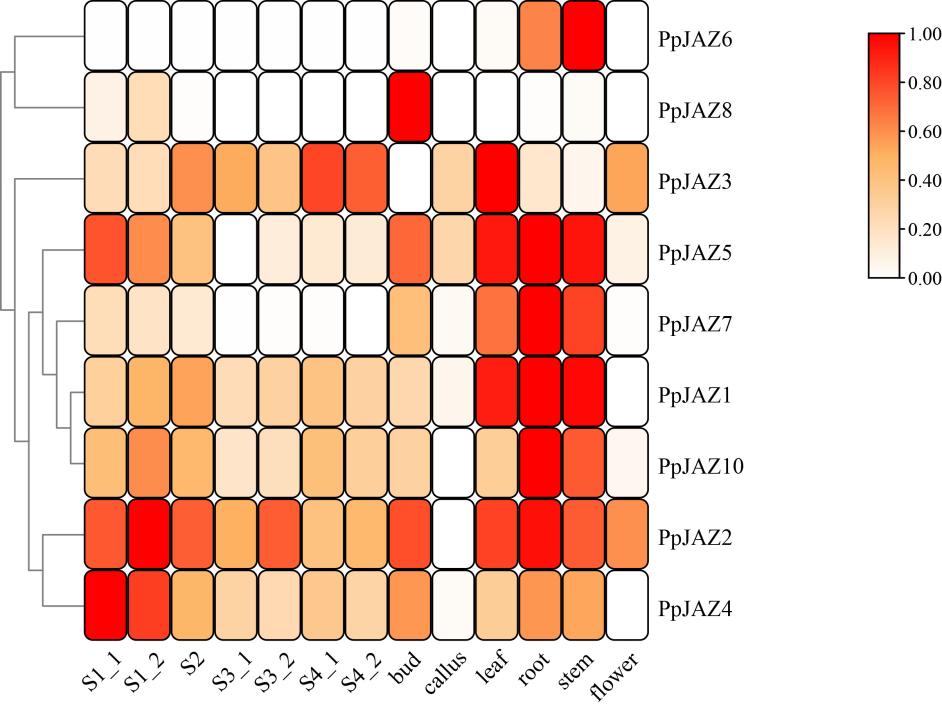


Figure S10. Tissue-specific expression patterns of *PpJAZ* family members. Data from PeachMD (http://www.peachmd.com/#/trans).


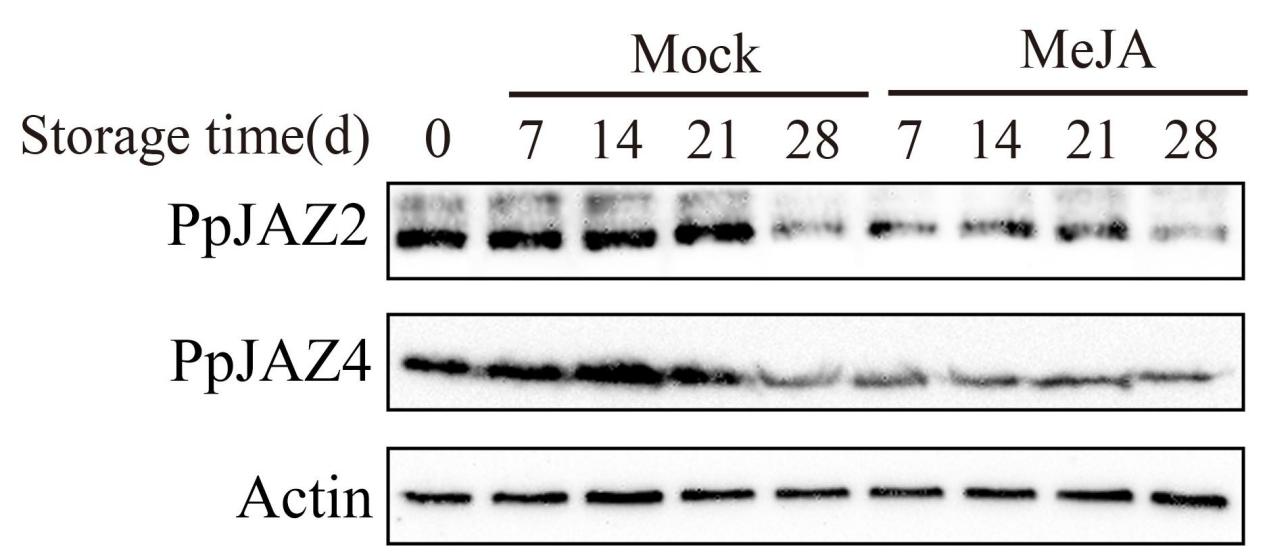


Figure S11. Impact of MeJA application on the protein levels of PpJAZ2 and PpJAZ4 in peach fruit during cold storage.


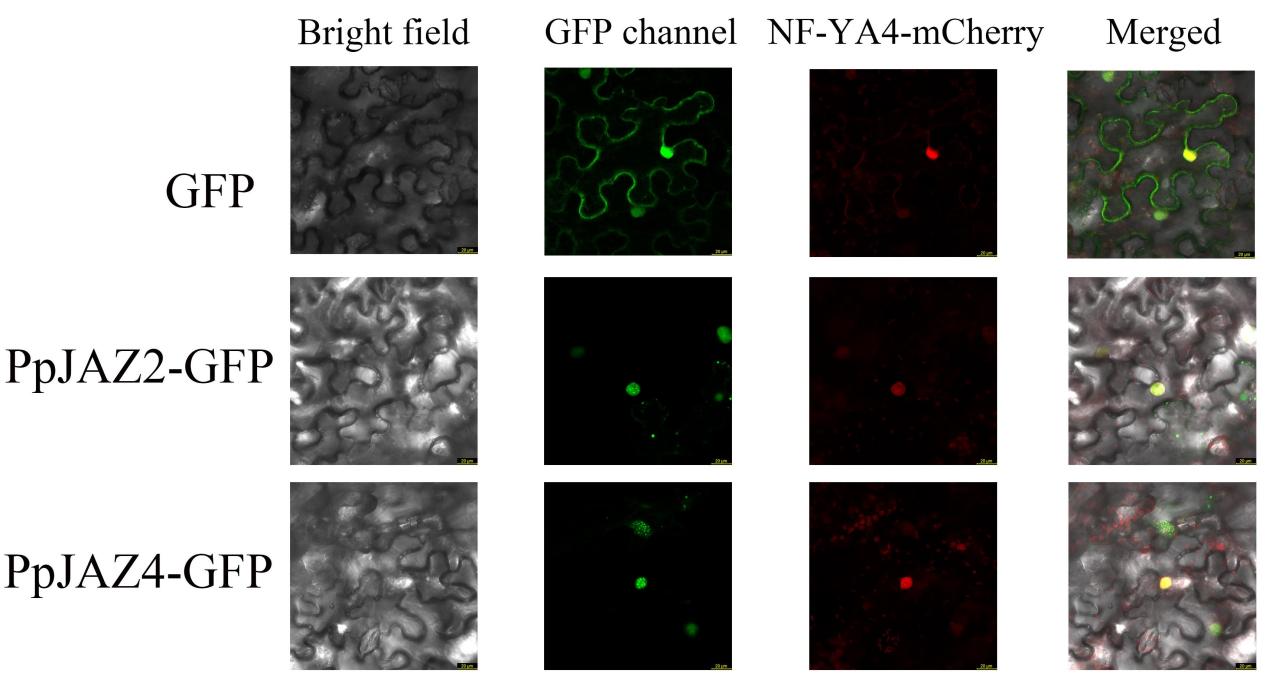


Figure S12. Subcellular localization analysis of PpJAZ2 and PpJAZ4.

Subcellular localization analysis of PpJAZ2-GFP and PpJAZ4-GFP in *N. benthamiana* leaves cells. NLS-mCherry was used as a nucleus marker, and completely overlapped with the green fluorescent in cell nucleus. Scale bar = 20 μm.


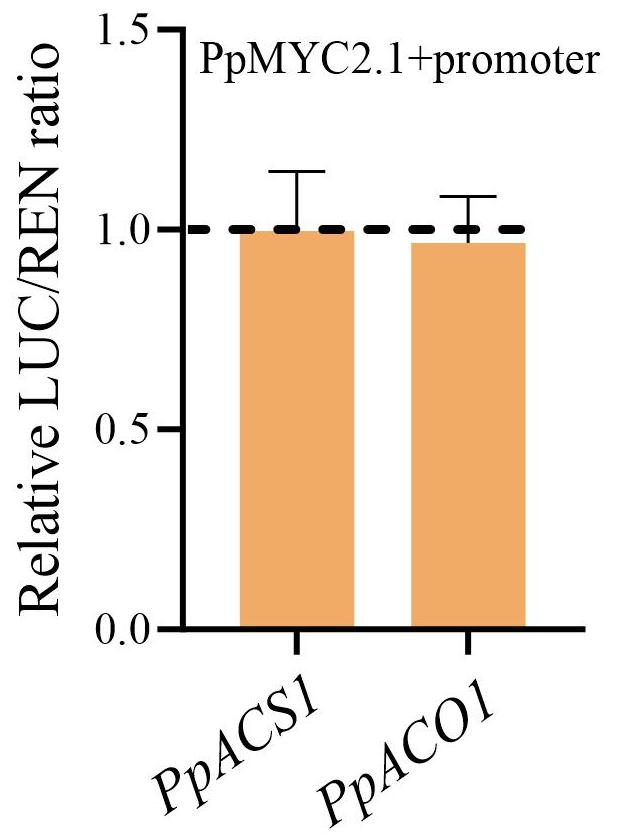


Figure S13. Transcriptional activation analysis of PpMYC2.1 on *PpACS1* and *PpACO1* promoters*.* Error bars represent means ± SE (n = 6 biological replicates).

Figure S14. Sequence analysis of the degron sequences in PpJAZ proteins.


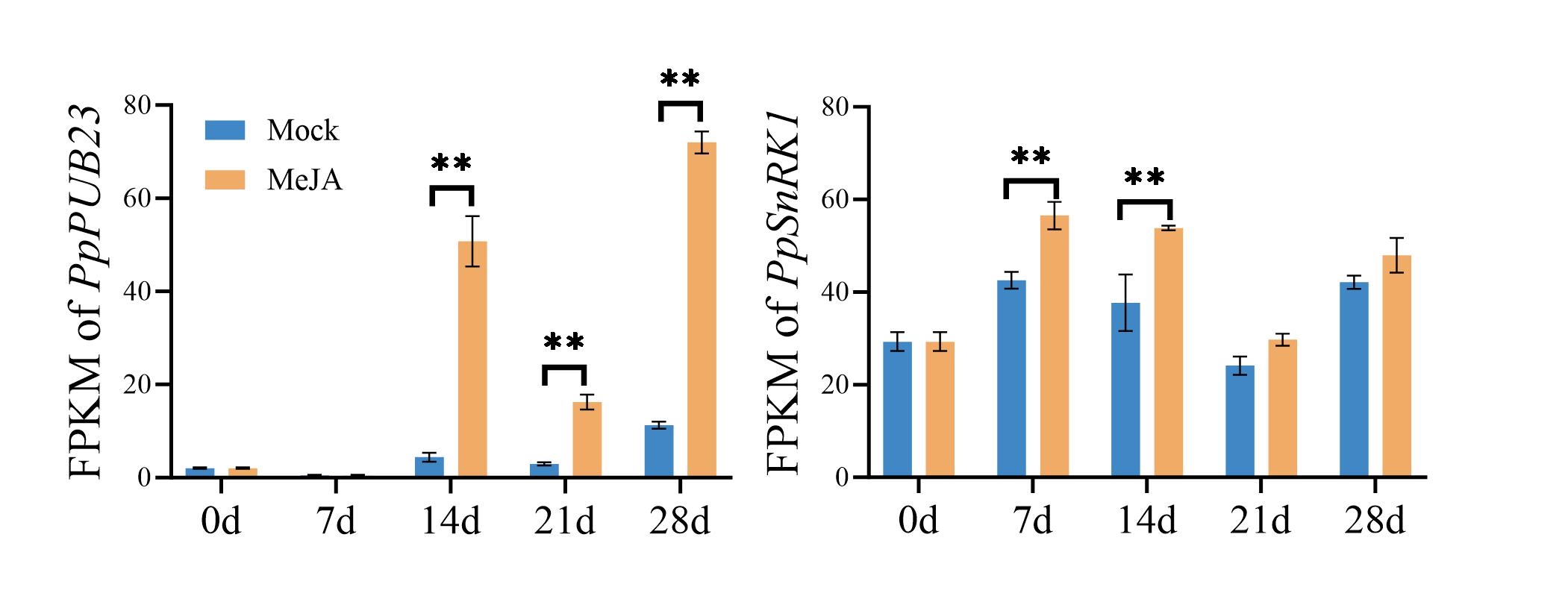


Figure S15. Effects of MeJA treatment on the expression of *PpPUB23* and *PpSnRK1* in peach fruit during cold storage.

Text S1. Amino acid sequence analysis of PpJAZ proteins. Red represents jas domain, blue represents degron sequences.

>JAZ1

MSSSSETLEVSGQRGLRMAEKPSSFTQTCSMLCQYLKEKGSFGDLSLDMACNMQQSNGTGTPEMFHQKAPPVNFFPFMENSRNLPATPGDFKSMDLFPQQAGFGSSVPRGDVPKMADSSVKKSVPGEPQKAQMTIFYGGQVIVFDDFPADKAKEVMLLASKESSHSQAAQASIPAKSNNVFASHLGKNPMNSSSSVPPSANMFPKFGNQVIQEAPKPSPQPIVCDLPIARKASLHRFLEKRKDRINNKAPYQTSSPAAGPAKPAEGKSWLGLAAQPTQ

>JAZ10

MNLVPLLRPFSLSPSYLEKNPLPPCHLSRSSNLPNIPPNKQFSKQFASMSRATVELDFFGMDQHRDPSSPSKSQFQKFLHRQRSFRGIQNAMYKIKPQVLKSVIASGSVLLNHHQHGSETPMASRKSFSVPSSPKAEQIPFPSLPVYIPTGTSVSSFMPAPAAAAAASEKLEETTTPLTIFYNGTVSVFNVPRDKAESLLKLALEGNSAKAAESALAVDSKLALHSSDQQQLLDPLDGDLPIARRKSLQRFLEKRKERLNSVSPFASHA

>JAZ7

MRRNCNLELQLLHPSYETHKKQQEQEQHEEQLQKQQQMMTIFYNGAMCARDVTELQARSILFLANREMEERVKSPFTPSGSELSSPTVMSPLCSPVAGMSMKRSLQRFLQKRKHRVQATSPYHH

>JAZ5

MAEKVNFAQTCNLLSQYLKEKRSLQVPTTMDLLTNMETGPAAETPSSKPSIDLFPQFAKNPEAVFSNQPGSAQMTIFYGGQVLVFNDLQAEKAREIMNFATKGSSKISSGFVSNGIDKFGSASVTKMVASEPNIAANSQDIQKVHSQVIGSDLPIARRASLHKFLAKRKERVAAIAPYQVNHQRASPSKSEEEMSSRDQVEGQCSKQLELRLY

>JAZ3

MERDFLGLSSKNGNLTVKEEANEEAKNSALPRSSGMQWSFSNKVSALPQFLSFKAPQEGGSRKTVHDTSAFMTISTADAFHFSQKPFSGVIQKNFTLDKQAGNHYAMTVSPVQQFDAHSVHHSQDQRIPIGFSSTTKLQPLGGVPVVAPVSLLPSKSSLVGTADLRNGSKSSGAPAQLTIFYAGSVNVYDDISPEKAQAIMLLAGNGPSPTHCKAPTIAQVPAPIPRPSPGDGVFRNQAHITSTISGLPSHLSVTSHASSHSGGAFSSTNELAIVKPVGTSASPIDHSEASKVVSSVGSAMTNLIPAVPVPQARKASLARFFEKRKERMMTTLPYNVSKKSPECSTPGSDGLSFSFNSSGSCPPQAIN

>JAZ2

MEEKSQAGDVKNTPEMEEKVVGQSQPMKTEEEAAKEKDPSSSHDLPNNSNTTRKIMPAQLTIFYAGSVSVFDAVTAEKVRELMLIAAADAANKTADVKNGGTSGPPSPLVCTGSSSLQNSAPGSPVVQPYPDQKSSICKLQAEFPIARRHSLQRFLEKRRDRLVSKNPYPTSPATQLDDDAKINLSNNASPGLGCFKQSAMVKEEMQPSSATAYPA

>JAZ4

MERDFLGLNSKESVLVVKEEINNDGCKDSGYARGAGGAHWPFLNKVSALPHLMPFKAAQDDKTKKMVSESFLSSGFMPISTADAFDHCQKQAPCEIQNYFNHDRQDGTHFSLTAYPMQHDVHSVHRPHDVKMISVTNQGFSVPVSNPFFKNPFATTGQNFAATTIKQQLQGIPVTAPYSVLPVSGSTEPWNNSKNSGSPSQLTIFYAGTVNVYDDISPEKVQAMMLLAGNVSSISSNAAQPKTQAPSAKLAVEDGVPVNQLTNTPPSGLSSPLSISSHTGVQSVSGSTNTDELMAPRTTGHPTSPVSKMEPPKIVNAVGSVAATSMIPSAVPQARKASLARFLEKRKERVMISAPYNFSKKSPDSNGVNFTQQGEQQ

>JAZ6

MACSTPIIIKDFFMKTDQELYGEDKNKSSSGFEKVARTPDDQQRDISLRSMDEENHMSQSVWFFRKYLLTRSQNNVAKTSMEAEFESEIIKGHSSPRPLPPKFLGSNNFPGHRLSLLEQQLLPGLRCDDQSNRTSEQLTIFYDGIINVYDNIPADKAQAIMRLASENSSVKPLVAESFKTDRQKPPLKPKSLSVSKIRAGLPMARRYSLQCFLEKRRDRNINNSPYALHSKKQEDNYEATVNNESNESDKLSLLPFPSRLGYFYPRLVNQGSC

>JAZ8

MPTPSPPLETPMRFQLASLFFPCIFLRGDWTLDQQQQITIFYNGQVFVSDITELQARAMILLAAGDMAERGISSVSNAALLALQSQIYGPPGVSMKRSLQSFLQKRKKRSQEASPYNSLQ

Text S2. Promoter nucleotide sequences of *SlACS2*, *SlACO1*, *SlPL1*, *SlPME1*, *SlXTH3*, *SlPAL1*, *SlCHI*, *SlCHS1*, and *SlMYB12*. The PpMYC2.1-binding motifs are marked and lined by red. Translation start site (ATG) was shown in yellow box.

>SlACS2(Solyc01g095080)

GCCCATGATAAGCCTCCATTCAAATGAAATATCAAAATCACTGTATTATTATAAGATACTTTGAGAATATATATTGTTTGGTCAAATAGTTTATTAACATATATATTATATATAAGTATGTGAAATGATGAAGCTAGAGTTTTATATGAACATATAATTTAGATTTTAAGTTGTATATTTTGCTCATAAATATAAAATTCTATGAATTGTAAAATTATCAATATTTACTTAATTCTTTACGCAATCTTACTAAATATATAAAAGTTAATAACTACAAAAGTATAATCATACGATCACAAACGAGCTATTCTAAAAAAAGTATCACATATTTAATATAATCCTCCCACATAGTACAAACAATCTTCTCATGTTTTGTAATAATAAATGATGTAAGGGTTTAAAGGTGGTGTGAATAATAATTGCAACTAAAAAATTTATTTACATCTAAAATAAATAATTAATACATATAAAATCGTATGATCAAAAATTTAAAATTTAAATCATGATATGTAATTAATATGTCCAGACACCTGCTTAATAAAAACTATACACTATTAATGCAGTATGCACTTTATACATATTTTGTAAATTAGATAATTAAATGGCCGGCTAGAGTAATGCAATACGATAGAAAAGCTCGATCAAAATTAATCACACTCAATGTGCCTAGTAAGATCTTCAAATCAAAATCAATTACGATTATCATCTGCGGTCCATTGTTCTCGTCCCTTCCCAGGAAAGTAATTATCCCTATTATATTTTTATTTATTTATATAAACTACTTGAAAAAGGTAAAAAGAATAAATAAATAAATTACCAGTAGTACCATTGTATTCTCAACTTTTTTCTTTCTCACGTGTAGCTTCTAGCTTGAACATGAAATTTCATATAACTATTTAGACGAAGGCAATTACGACTAAGGGTATGTTCGATAAGAAAAGAAAATATTTTCTTAAAAAATAAATAAATTTTTAATTTATTTTTCATATTTGATTAATAAGCAGAAAATATTTTTGAGGAAGTATCTTTTTTTATTTTTGAGAAAATACTTTCTATGAAAATAATTATTGATGTGAAAATCAATCTCGATAATTGTTGCAGGAAACGACTCTGACAATCGAATTAGGATAAAACCTCGATGACCTTTAAAATCGACCCTAAAATCTGATCCAAAACTCGATCCAGACTTCCGATCCAAAACTTGATTCAAGTAAATATTTTTAAAAATAAATTCTTTTTGACAGGGTGGCGTAAAAATAATTTTATTTTAAAATATGATATAGTTTTCTAAAATATATTTTTTTGTTTGGTTGTTGGGGGTTGGTTCGAGGCTAGGGGTAAAAATAATTAAAACATAAGAAATTTTAAAAGTTTTAATTGCATTTTTTTTGTGTTGGGGAGGGGCGGATTTTGGGTTGGATAAGAAAAAATATTTAAAGATAAAATAGAATTTTGGAAAATATTTTTCTTAATTTTTGAAGGAAAATCATTTTTCTTAAATTTGAGAAAAATGAATTATTCTTAAAAAAAATTTCCAAAAACATTTAAGCTACCAAATATGAAAAAATAAAAAATATTTTTTTTCCTACCAAATGCACCCTAAATTAGTCAAATATCCAACATTTAAAAGAGCTATGAAAAAAAAAAAGAAGTAAGAATCGTAGATCTTCTTTTAATGCGTACTTTTATTTTCCAAGATTTGAACAATAAAATAGACTTTTCTATTTTTATTTTCTGATGTAATTCTTATATACGTTAGTCGACATGTTCTCATTACATACTTCAGTCTTTCCCCTTATATATATCCCTCACATTCCTTAATTCTCTTACACCATAACACAACTACAACAAACACATAATACTTTTAATACAATTAGTTATTTATTAGAAGTATTTAAAGTAAAGCACTTGTGAGTTGTGTACATTTTATTAATCTTCATCTTCTTAATTCTCTTCAGTTTTTAATTTCTTCACTTCTAAACTCATTTAGTAAAAAAAAAATG

>SlACS2(Solyc01g095080)

AGATGAAGACTTAAGTACATTCCGAGTCCAAATACATATTCAAAATTTAAATTTGATAGATTCAGTTTTTATGTTTTTAGTGCTGATTACAACATTGAAATTCTAAATTTAGAATTTAATATTTATTAAATGTTAGTGCATTTATACAAATAACATATTACATCTCAAATAATATTGAGTTTGTTAGATTTTATTTGCCCTGATTTCTTATCATAAATAGGTTTTCCTTTTAGGAAAAGGTTTTGAATTGACTATTCTTTTTTTGGTAGGAAAAAGTTTAGGACTCTATAAATAGAGGCATGTTCCTTCTAACTTAATTAGCATTCACAATGTAGTTTTAAGGGCTTTGAGAGTTTTGGTTAGAGGGAGAATTTGTGAACCTCTCATGTATTCCGAGTGAATTGGTTGAGGTTGTTTCCCTCTGTATTTTGTACTCTCATGTTTATAGTGGATTGCTCATCTCCTTTGTGGACGTAGGTCGATTGACCGAACCACGTTAAATTTTTGTGTCTTTTGGTATATTTCCTGTTCTTCTTACTCGTGGTCTTTCGAGGTTTGCTTTGCTAGCTTCCGCGTTTACACCTGCTTATTTTCGGTCCTAACAAGTGGTATCAGAGCCAGATTCAATAATGGAGTCAGGTGTAGTGGTTCGATAATCGATGATTGAACCAAGTTAGAAAGAGGTGTTCATCTTGACGGGTGTAGTTCTAGCCGCAACCTTTTTGACAGTAATGAAGATTTTGATGGAGAAATTGTTTCAGAGAGGTTCTCTGTGTTGAGACATAAATTTTGTAAAGGAGATTATGGAGAGGAGAAGCAAGTTGTTGAAGATTAAGTAAAGAAGGTGGACAAATTTATTTTGTCAGAAATTCAGGCCAAGGGGGAGATTTGTTGGGTTTTATTTGCCCTGATTTTTTACCATAAATAGGTTTTCCTTTTAGGAAAAGGTTTTGAATTGACTATTCTTTTTTTGGTAGGAAAAGGTTTAGGATTCTATAAATAGAGGCATGTTCCTTCTAACTTAATTAGCATTCACAATGTAGTTTTAAGGGCTTTGAGAGTTTTGGTTAGAGGGAGAATTTGTGAACCTCTCATGTATTCCGAGTGAATTGGTTGAGGTTGTTTCCCTCTGTATTTTGTACTCTCATGTTTATAGTGGATTGCTCATTTCCTTTGTGGACGTAGGTCGATTGACCGAACCACGTTAAATCTTTGTGTCTTTTGGTATATTTCTCGTTGTCTTCTTACTCGTGGTCTTTCGAGGTTTGCTTTGCTAGCTTCCGCGTTTACACCTGCTTATTTACGGTCCTAACAGAGTTCGATGGGTTGAATCTATAAAAAGAAAAATATACTCGTGATTCACGATTATTTATATGAAAATATAATAAATATTGAATTTCCTTTGCTATTTCTTATGTTTACGTCTTTATATTTCAAATTATTCCACCAATACTGACAAGCCCTAGGCCATCTCTAGGAAATTCATACAATTTTTTTTTTGTTGTTAACTAGTTAAATTGGCAGCCTTAAAGATTATTGTAAAATTCAAGGCAACTTCCTCAAGTACTACAACTACATTGTAACATCCCAGTCAAAGTGTCCTAAAATTTTATAAAATTTGACACATGAAACAATAGCACAATAAATTTTAGTACTATTGCAGCCATGGCCCATAAGCCATCATGTATTATAGTCAAAATGGGTCCTTTTCCAATTTGTCTTGATCCCAAAATCCCTTTGTAGGTAAGATGGTTCAACAAGGAACTATGACTCTTAAGGTAGACTTGGACTCATAGACTTGTCATAACTCATAAAGACTTGGAATATAATAATTATTCATTTAAATTATAATTCTCTACTTTAATATCTTCTACTATAAATACCCTTTCAAAGCCTCATTATTTGTACATCAAACATTGATATTCATCTCTTCAATCTTTTGTATTCACATATTCTATTTATTCAATACACTTAGGAAAACACTTTACCAAGAAATTAAGATG

>SlPL1(Solyc03g111690)

CATGTATCGATATATAGTTATCATATAAAATGCAGCTGTTGGAATATTATTAAAATGATCAAAATCATATACGGCCACTTAAACTTGCTTCGATTATTCATTTAGACATCTCAACTAGACGTACTACCTATTGAACACTTCAATCATTTTGAATTTGAATCATTTGAACATTTTTTCGAGAATAAACAAAAAATAGAGGATGTGTGAAATCACTCGCACTGACATGTCAATTTGACCAATTAAATAATGACATGTGTTATTTTTTAATCCAAAAAATTATTTTAACATTATATTATAAAGTAAAAAAAAAAATTATTTTAAAGTAAAAAAAATGAAGAAAAAAAAGTAAAACTTAAACACCTACTGCCTTCAACTTCAACTGGAACAAAACATGTATTTCCTCTAACTCCATTAATGGCGATCCTCCCCTCCCTGTACAGTTCCATCTTTAACCCGACAATTTTTTTTCATCAGCTTTTTTATTAATTATAACTTTTTACTTCATCTCGTTTTTTAGGTAGAGAAAGATGCGGAAAAAAATCTCTCCAAAAAATTTATAAATGAACATAAAATGCTAAGAATTTTGAAAACATTAGAATGGTGGGGGAAGAAGATAACCACTGATACTAAGAATTTTGAAATCATTAGAATGGTGGGGGAAGAAGATAACCACTGGTGTATAGGTATGGGGTTGGGGTGGGGTGGGTTGGGGTTGATAGAAATAGGGAAGAAGAAGACAACTGTTCTTCTTTTTTGTTAATTTCTTTAAAAAAAATTAACTTAGAGGTATAAATTAAGGGGAAAAAATAAAAATATTATTTTTAATAAATTAACGCGCTCAAGGAAAGTGAATTACACGTTTTTTATCATGTCAGCATTTTGTGTCTAATAGGAATGACTTTTGAACAAATAAAGTGTTCAATTGATACAAGGATTAGTTAAAGTGTCTAAATAAATTATGCGGACAACTTTGAGTGGCTGGAGATGACTTAGGCCTATTAAAATAGACAATTATGTATAGATCATGCATGCAACCCTTAATTATTTCTACACATTATTCTATGACATAGAAATTACAAATGATTTTGAATTTTTATTTTTTTATATATAGTAACTTGCATGCGGGAAAGGAGCAATATAATATGGCTATGGTGATCAACTAATTTATTATTATTATTAAAAATTGTATTTTGTTCTAAGAAAATCACATCATTGCAATAGCCGATATCAATAATTTTTTAGTTCAGCGCTATATACTGAAAGAATATTTATAAAATTTTAAAAATTCTCATTGGACTAATTTTGTATATATAATTTGAAAATATTAAGAAATGAACGTCAATATTTTAAAAAAATTAAGCGTATTTTGACATGACACTCAAATTGGACTTTGTTAGGAGAAAATACTTTTCTTTGAGATTTTTAAGCTATGCTATATTAAATGCTATAACATTTCATGTCATTAATATATATTATAGCAAATAAATTATCTTAACAAATATGAGTTATATTTTACAAAGTCTCATGTTAGTAAATATTTCTTGGATGACCTAATAGTAAAAAGCTCTTTAAATCAACAACGTATATAACCTAAATACATTTGATTGATCATGTATATAACGTATACACGTTTAATTGACATATACCAATCATATATCAATAACAGATAGTAATTTTACGTAATGATATTGAACGATGTATATATATATATAACTTAAACGCGTTTAATTGGACGTATGCTAGAGTAGTAATCATATGCTAACAAGAGATAGATACTGTTACTTGAACAGACAAAAACTACCGTAGTCTTCTTCTCTCTATCATCTAGGTTAGAGGAATCTCCGTAAAACATGTAATCACGAAGGAACAAGGGACCCATCCCTTTTGTTTAATTACGCTAATTTCTCTACTTTTTCCTTCTTTATAAACTCCTCCATTTCCCTCTCTTTTCTTCATAACCCAAAATCCTCTGTTTTTAAAGCTAATTTATAAAACAAACAATG

>SlPME1(Solyc07g064170)

ACTAAAAGTGCATAAAGTAAAAATAACTATCACACAAATTCAATATTCCAATAAAGATATTAAAATACAACATGATAAGTTAATGTATACAAAGCACCAGGCCATAGTAAATTATCAATAATAATAACACAACAATAGAAAGTATGTTGTACAATAACAACAAGCAAGAAACATAAATGTTAAACTAAATAAATAAAAAGAGACAAGAAAAGAGAGCATAGAGATTTTCTCATTTTTTTAAATTGGTTTAAGTCTATATATTATTGTGTGAAATGTCACTATTTATTATTGGATAACATTGAAAAATACAAAGAGTTGTCATAAATAAATATTAATCTATTTGAGATTATCGATGAGAAATGAGAGTAATGAACATAATAAATATAATTAGTGAGAGTCACGTACATTTACAAGTTGATGGTAAGTTATATATACTAATATGATAATTTACTATTTACTATATTAATATTAATATTTAATTTATTAAACAATTTATAAAATTTAAATGTACAGTGCAAATAAAACTGTAATCTAAGAAAAAAATATATAAAAGAATTCAAATATACTAAATCTGTAATGGAAAATCATTATGACTAAGTATTAAAAAATCAAATACATTCTTAAAATAAAATTACAAATTCAAAAAAAAAATCAAATATGGAAGCAAGTTTAGCATAAGACTACCAACGAAAATGCAAATTGATAATGTAAATTGAAGACATTCCTTTCCCTATTTTTAAGACATTCATTTCCCTGTTTTTTATTGATAATGCAAATTGAAAAAAAAGCTATTGTAATAAACATTCAACCTTTGACCATAAAAAAAGGTTAAATGTTTTGGAAGAGAAGTTAACGAAAACGACATTCAACTACATAAATGGAGGGGTCTTATCATAGTTCTAATCTCGGCAAATTACAAAAACAAATACATCAAACTCCGCTATTATAGTAATTGAAGTGTTATTCATCCCTATAATCACATTACGCCTTGTAATCTTGACAGAAATCCTCAAAATTTTCTTTACTTGATTAAAGCTTCTCTAGTCTTCTACACACAAAAAATGTATCATGTTTAAATCTTTCTATGAAAATACTATTAAAAAAATTTCATAGTATAGGGAAAAAATATAATTTTGGAATATGAAGATTCACTTACAAAGTTGAAAGGTCAAGTATTATTACAAATTAAAGACAAATATTAATTAAAAATATAAATTAATTTGGAAGATGAAACACATACATGTATCTATTCAAAGGAATAACTCTTATTTTTAATTTTTGTTCTATTGAATTTACTCATATGTGTATACCAATTAAATAAAAATATTATAGAAATTCTACTATTTACTATTTAATTATTTATTTAATAACATTTGAATTTAGCATAAGGGTAAAATTGTAATTCAACTTTTCTACTTTGAAGCTTCCCACTTATAATAATATATGATTGTTACATTTTATTATTCATTACAATTTATATATATATTTCCATGAGATTTTAGTTATTCTAACGTATCTATAAAAATTCACATGAAACACACGTGTAAAGCACGTCCTTAGAAACTAGTATATAGAAATATAGATATGATACGATGCTATTAAGGTAATTTCTTCCACCATTTACTGGGAGGGTAAATCACCAAGGGATATGATAAAGATGAATTGGATTTAATTTTAATCAAAGATTTCTAAGTCCAATTCTATTAATGAAATAATTACCCCTTTTTAAACATAGCCTAAATACATTTAAATCCCTTAAATTTCATTTAGACATTCAAATTATGATATGTTACAAATTTGAACGCGGTCGGTGGTCCTAAAATACACTTTCAACTCAAATTTTGAAAAAAACTTTTACGAGTATTTTCAAGCGTCTATTATTATCTAGGTTAAAGTTTGACCGCATAAAATACGTCATGCCTCATATTATATAAACAAAGTATTATAGTAAGTGAAAGTGTATCAAAACACTCAACACACATAGTTTGCAAACTTCTAAAATG

>SlMYB12(Solyc01g079620)

GTCCAAAAATTATCACAAAGATTTATTATTATTATTATATATAAATCATGATTCCAAATTGAAAGTCACCATTTTGAGTATGGTTAATTTAAAAGAAATATATTTTATAAAAATAATTCTTAATATTCTCATGTTCAGTTGGTTAAAATATTTTAATTTTTTTTTAAAAAAAAGAGAAAAATGAATTATATGATAAACATAGAGAATACATTTTTTATAAACATATCGAATTCATTATCTCTATCTCGTCCAACTAAATGACCGCCACTCGCAAACCCTCGCTACTTACCTTTTTTCCTACATATTTAAATAGTTAAAAGAAAAATTATTTATAGATAAAAAAAATTCATTAATTTTGAGAATTATTTTGAAAATAATATATTTTTACTTCATATAATTTATTTATTTTTATATCGCACGTACAAACGCAAACTAAACAAAATAATGGGGTGAAGGACAACGAACGTGGTAAATAGAAGCCCCCTGGCAGTAGTCGACTGTGGATGAGCCACGACTATATTTTAAAACAAATGGCTACGTGGCATATTTTAACACCACACACGTGTCCCTCTGTCTCTTTCAATATCTTTCATATAAGAGTTAAGTAGCGAGGACAAAATTTTTATTAATATCTATAATTATAGCTAAAAATGAGTACGAAGCTAATAGGACTAAAATTCATGATTTTAAGAAATTTTAGTAAATTCCTTGATTATTAGACTAAATCGTTCTAGAATTGTAAAAATTTATATATATATATATATATATATATAATAAAATTTTAGCTTTATATATGATGAAATTTTTTCGGCAAAGGGATTGGACAAAGGGTGGCTCTGCCCTTGGATGTTGCGTTTTTTAAAATTAATTTAGTTAATTTCAAAATTAAATTAGGTTATATTTATTTGATACAAATTATATAAAATGTCACATAATTATATTTTTTTTATATCAATATATAATAAAAACATCTAAAAAATATTAATTAAATTTCTTATCATTTGACTCTAAGAAAGCAACGATGTTGATCGAAAGGGAGCGAATAGAGTACGTTATTTGATTTTTAAATAATAATACTCGAGGTTTGAAAATAAAATTTTAGAACAATCGAAATTGAGGGTATCGAATATGTTTAACCTTAAAACGGTACAATTATTTAGATTTATAGACTTCAATCAAGGACATATATAAGGATTAAGATAGTCACAAACACGTGATTTGTATACTATATTTTGATTAATGAATGGGCAAATTAAAATGAAATAACAAATATATATTTATAACGAGACATAAATACGTTAAGTAAATAGAATTAGTTCTGAAATCCGATGTCGATAGCTAGCAACGTCATCGAATAACCTTGATTATATTGGTATTAATTGAAACAAATATAACGTAGAACAAATTAAGCTCAAGATCTTAAAAACACATAAAGATATTACATTACTACAAATGAATTAAAAAATGCGATAATTTACAATGAAGGAAAAGGAATTTTTTTATTAAGTAAAATCATAGAGTAATCACCACCCACTATGACTCCCATCTACCTGGTTAAAGAAAAATTAGCATAAAAAAGTCTTTTATATATATATATATATATATGAAGCAAAGTGTTCTAATTATGAATAAAGAAATATTTATTAGATTATAACGATGATTATATTTAGGATGGAGCTAGCAATTTATCAGAGGATTCACCTCTTTTAATGAAAAATATTATTATCTGTACATAATTAAAATGATTTTTTTTTATAATATACAATAAATATCAAATTCCCTTCAATTATTTTATACATTTATTTTTTTAAGTTTTAAATTTTTTTTTATTAAAAATCTTAACTCTTCTTTTATCAAAGAGTGACATGCAATGCAAAAAAGCTTATTAAGTCAACCTTTGGTACGTTATTAAGTTCACATAAAATTAACTAGACTAAAGTGAAGAGCGGGGTCCATTTATTTGTGTTGTCTCTCTATTTATTGGCATTTCTATTGGTGAAATG

>SlCHS1(Solyc09g091510)

CAAACACCACACAACATACCTACTTAACAATCAATACAAATACAGTACATTCTCCTAATGGAACCAAACCAAAGAAATGTTTAAATTTTTGTCACATTAGATGTTTTCCTATATATGTTGTGTACATTCTACTTACCTTTCATTTTACCAAACATCCGTCCGAAATTTATTGACCCGATTAATTTGAATTTAATTAAACAATAGAAAATTTACGATGAAATATTTTAGTGTGTTGATAGTTAATGATAAAATAACTATTTCACGAATTAAATGTATGATATCTAACTAAAAATAATTTATTTTTTTTACCGTTTGATCTCTATATGTCCATCAAATTTTACTTCATTCATGTACTTCCCCCATATATTAGGTAAAACTCTAACTTAAATAATATATGACCAAGCATATTTAACATTTAACCTTTAGGTTATGAACATATTCTACAAATTTTGTAATTACTTGTGTATTATAAAAATGTATAGTAGCTATAGGTTGTTAATTACGTCATATTTAAAAGTAATTAATGTAGTCTAAAAATTAGTTTGAATACAACATTTTTCGACATCAAAGAATATTCAACATATATTTTTTACAAATTGAAGTTTAAATATATTGAATCATGGAAATTAAAATTAAAATTATTTTGTCAAATTATAAATAAATCAAAAGTTCTATTATCTCCTTAAAAAACAAATAACTATCTGTTATTTGAAACTCTTTCAAATGCTCTTGAAATTTTTAAAATTTAAATTCGATATATCTAATTCTACACATAATTCATTTCATTCATGCATCTATCACTTTTGCTAATAATTTTTTTTGTGGCCACCTCATAAGACTCAAAATGTGAAAACATATGATATGTTTTTTTCTGCATTCATTGTTTTCCTAAAAAAGATGAGTTAGAATTATTATTTTGGAAAGTGCTAAATGATTTAGAAAATTTAATCAAATTTTGGCTAGAAAATTAAGAAAATAATACTATTTTTTAAAAAAGAATAAATTAATATTTTTTTTCAAAAAGTAAAATAATATAGATAAAATATTATTTTGATCAAATTTTCTGGCCAAAAAGGTTTGCCTATTATATTTTTAAGAAAGAAAAAAATCTACTTAATTTATGTGTAAATATACATATTGACTATTTATTTAATTTGGTAATGACAAACTTATGTTCAAAATTTCTGTAAAATGTGCTGTAAATTGTTAAATTCTACAGAATTAAATTCTCACCTCATAAGTCAAAATCTCATAATTAACTTACATGTCCAAAATTTCTTGTAAAATATTAAATTCCACAAAATTAAATTCATAACACATAGGTTATAATGTCATAATTAAATAAGAAATATATTTGATTTTTCAAAGAATATTACGGTTCGAAGTTGAGGTATATCAGTTTTAAAGTCTCTTTTATCATAAAATTATGCTATATATACAATAACGACACATGTAATATATATAATCCCACAAATAAATATAGATCTTGCTCCTACCTTATGAACCATTTCGAAACTCCGAACTTATAAAGATTGAGAAGTTTAATCCGTATCGAATTTACCCTAAAAAAGTTAAAATTCTTCATATAGTGTTACGTGTCAACTTCTTGTCCAAAAGCCAAATGTATATCAAAGGTGATGCAATAAAATAACAGAAATCTTATGTAAGATTTCATCAACACCGTCAATTTTTATTTCAATATAATTAACAATATTTTATGATCGTTGATCTTGATAAAGTAGGTAGCTGTCCAATATTTATTATTATTTATCTTAAATTTAGAAAGGAAAACCAAAAAAAAAAACACAAAGTCACGTGACATTAGTTGTTGAAGCACGTGATCTCTAGCTACCATTCTTTTTCTTTTGGACTTCATATATAAATATATAGTTCATACAACCCCCATGCAACAAAAATACACCAAGACAATTATCACTCTTTCATTCACGTAGTCCTAAACACAAAAAAACCTAGCATATCCACCATTTTTTCCGGCGAAAATG

>SlPAL1(Solyc05g056170)

GCAACAATTCATTCTCAATGCAAGTATTAATAAAGAAAATGTACACATACAAAGAAAAAAAAATGAAGCAAATATGGTGAAATTTGAGTCAAAATCTTAAAAAGAAAAAAAATGTTATGGTAAAAGATGTAAATAAAAAAAATCACAAAATAAAACGTTAAATATTTTATACATACGCATTTAAATAATAAATAAATAATAATTCAAAAGATAAAGTGAAATTTTAAAATCATCATAATCGTATATTCAAATTTTATTACGATACAAATTTGGAAAATAATCGGCATAATGTAACAACACTATATTTTTGTCCAAGCAACTAGTACATATATTTATTAGTATATTATAAAGTTTTGTTGAGGTACTACATTCCAACTCATAAATATATTATATATATATATATATATATATTAATTAATTAATAAATGATATAGTAGGTTGCCAATAAAGTAGGTAACTATATGAGGGATGTGATAGTACGAAAAGAATTATTTCATTTTTAATCAAAAATTTGGATTTCGAAAATTAAAAAAAAAAACTTACTACAAATAGCATTTTTTATAAGCTTTATTATTATTATGTGAATTCAAATAAATCAAGACTCAAAAGTAGATAATATCGAATAATGTGAGACAAAATAAAAAAAATACTTTTAAATGAATAATCTCTGTCACACCTTCAAACCCCACATTATTTATATAACATAAATATGATTTCTCAACCGCTTCATTCTTAATAAAAAGTATATAATTTAGATTTCGGATATAAAAAAGATCATATTAGGAAGCATTCCCTAATTAGATGAGTCTTGATCAATTAGAATTTGAAGTAATTCGAACTCCAATATAAGTATCAGATTATATTATAAAAATCATCATTTATGATTATTGTTTCTTTTTACATAGAAATTTTTTTATAATTCCAAAAATAATAACTTTATGAAATATTCTTTTTTGTACATGCCTACTAATCAAAGTAAAAAAATTTAATACTAACTCCTAAAACTTCTCATTTTACATATTCCTCAAGTATGAAATGACGTTTCCTTCCCACCAACCATGCAATTTACAATGAAACTTCGTTTTCAATTTCGAACTATAAATCATAAAAATAAATCTAAAATTTATATATTATACTCAACCTAATTAATTATGAAAGTTCAAAATCTTTTACTTTTATTATTGAATTGATGATTTATTTATCGATCAATTATCCTTTTTGAGATATCATTTAGGAAAATTATCTTATTTTTTAAAAAAATTCTCTGTAATTTATGAATTATGAACTGTATTTTAAACGAAGATCTAAAAATCTTAAATTTTGTATTCATAAGTTATTGCAATTGTGAGATGACAATCTAATGTTTCATCTACATAATTGTTCATTATATCAACAAATATTATTCTCAAAGGTAATTTCCTTAAGTGTTATATTTCATCAAAATTAGAAGTAAATACTTATCTAGAAAAAAGATATCCATTGGGATCAAATGAACCCTTTACTTGTTCTCTACTATTTTTCACTTAATATTTGTTTGATATACATAACTTCATTAAATTTAAATTCGTATCGCGTATTTTTTATTCCATTTGACTTTAAATTTGGTTGGTGATATTTTTTTTTTTGTTTGTGTTGTTGTTAGTGAGAAGAAGAAGCCTATACTTGGCTGCCATTCCTTCAAAATTTCTATGTACAACCACCCTCATGATCTCCACGTGTCACCAACCGTAACCGTCGATTACCATGTGCAATCCAACGGTCAAGATTAATCTTCAACTAACACATTGGCCTGCAAAGTATTATTTCCTTTTCTATTTAAACACCTAACATTTTGGCTCAACAAACTCAGGAAATTCAGCTTAATTTATTGCTCATAAGTTTTGCAATTTCCTAATCCCAACAACCAAAAATATTGATATATACATTGCACCTTTTATCTCAATTATTTTATCAAAATAGTTTGTTTTCCCCTTGTTCAATATTGTTTTACGATAAAAATAATG

>SlCHI(Solyc10g055800)

ATATATATATATATATATATATATATATATATATATATATATATATATATATAAAACAAATTGTGTTTTTAACATAATTACAAAATCTAATTAAAAGTTATTAAATTTATCCGGAAATATATCAGCTCTAGCTTTTAAAATTTAAACCCTCTTATTAATACATAGAAAGCTAGCTAGCTACAGGCGAAAATGCATCATTAATAAATAAAGTATTTAGAAGTTAGATCGCAAAGCAAAAGAGATCTTGACCACAATGGATATCTAATTGTAATACACTTTAAAGAACAAATTACCATAGTATATGAGACGGAACTAAAATTAAAAGTTTAAAATGTTAAATTAATTTTATTTAGGAGTTCACAAGTTATATATATATGTTTATTTATTTTTCATATAAATATACGATTTAAAAAGATTTTATAAATTCGTTTGAACATATAAATCTACCTAGCTAACTCCATTTCGAAATAGTACTAGCCACTGAAGATATTATACTACCTCCGTTTTAAAAAGATTGATATAGTTTGACTTGGAACGAAATTTAACAAAAGAAAGAAGACTTTTTAATCTTGTGGTTCTAAATTAAAGTATGTTAAATATATCAAAATGCTCTTTAATCTTGTGGTCTTAAACATGACGCGTTGAAAATCCTTTTTAAAATAAACTAAAAAAGAAATAAAAATATTATTTTTGAAACGGAAAGAGTATGATTGACTTAGATTTTTTAAATACAAAAGAGTCGTGAAGGGCTTCTGTAGATTTGATCAAAATCATATAGTAATATATATATAGAGAGAGAGGGGGGAGAATTGATCAAACCAATTATTTAAGCATCTGAGCAAAGTCATAAACATATAACAGTCTCTGTTCCATGTTTGTGACATTTTTTATTTTATTTTTAGAATCAAATAACGTGAATTATGATTAATATTTTAAAATATATTTTTTATTATATTTACATAAAATTAATTTTATCTTTTATATTTTTTATATAATTTTTCAAATGAATAAGTTTTAATCTTAAAAATTTAAACTAATGTAATTTTAAAATTAATTAAATGAACTTAACTCGTGCTATAATCTTAATTTATTATGTGTTCCAAGTGAAAAAGTACCTTTTTTAATATTTGATTAACTTTAAAACATATGATATCGTTGTTGACTCTTTATAGACAAGCTACATAAAAACCTTTTTTTTTTGTTGTTGTTTCACCCTCTGTTTGTGTTTATTATTATTTTTGTCGATTAGATGGGCTCATGCCGCGTAAAATTTATTATTAATTAAAAGAAAAGCATTTGAAAAGTCTGGAAAGTTAAGAGCCGCCACTATTTTTTAATAATTTCCTAGAAATGAAACATGAATAGTTCTAATAAATAAAATAAGAAGCTAATAGCCGCCGCTTATTTATTTATTTATCAAACTTTTTTTAAAATAGATTTCTGCTTTTAATCTATTAATTGAATAAAAGACCATAAAATTTTTTTTCTCTATTATATAATTAAAATACTTCCAACATCCATATTTATTTGTTGCTTTTCAAAAATTAATTTGATTAATTTATAAAATTATAATTAGATTATATTAATTTAATATTCTAAATAAAAAAGTTTAAATATTCAAAAGATACTATAAATCATAATTTTTTAAATATTAATAGGATGAAAAAATTAAATCTATACAAATCTTAATAGTTGGACTATTAAAAAGAAAACTATAACAATCATAAATAGTTGAAGATGTATTAGAGTAACCAAGTCAATAGTTATATTTTCATGATTAATTTGACAAGTTTATATATAACAAATAAAAATAAACGGAATATATATTTATTTGATGCGGATAATAGCCGCCGGTATAGTATACTTGATGATCCGGATAAAACAGAGAACAATGAGAAATTGAGAATGATGTCTTGTTTAGTCAACATCCTCTTTACCTATAAATAAATACCTTATACTTCACCATTTTCTTCCCATCACTTTTTACTATTTCCATTAAAATG

>SlCHS1(Solyc09g091510)

CAAACACCACACAACATACCTACTTAACAATCAATACAAATACAGTACATTCTCCTAATGGAACCAAACCAAAGAAATGTTTAAATTTTTGTCACATTAGATGTTTTCCTATATATGTTGTGTACATTCTACTTACCTTTCATTTTACCAAACATCCGTCCGAAATTTATTGACCCGATTAATTTGAATTTAATTAAACAATAGAAAATTTACGATGAAATATTTTAGTGTGTTGATAGTTAATGATAAAATAACTATTTCACGAATTAAATGTATGATATCTAACTAAAAATAATTTATTTTTTTTACCGTTTGATCTCTATATGTCCATCAAATTTTACTTCATTCATGTACTTCCCCCATATATTAGGTAAAACTCTAACTTAAATAATATATGACCAAGCATATTTAACATTTAACCTTTAGGTTATGAACATATTCTACAAATTTTGTAATTACTTGTGTATTATAAAAATGTATAGTAGCTATAGGTTGTTAATTACGTCATATTTAAAAGTAATTAATGTAGTCTAAAAATTAGTTTGAATACAACATTTTTCGACATCAAAGAATATTCAACATATATTTTTTACAAATTGAAGTTTAAATATATTGAATCATGGAAATTAAAATTAAAATTATTTTGTCAAATTATAAATAAATCAAAAGTTCTATTATCTCCTTAAAAAACAAATAACTATCTGTTATTTGAAACTCTTTCAAATGCTCTTGAAATTTTTAAAATTTAAATTCGATATATCTAATTCTACACATAATTCATTTCATTCATGCATCTATCACTTTTGCTAATAATTTTTTTTGTGGCCACCTCATAAGACTCAAAATGTGAAAACATATGATATGTTTTTTTCTGCATTCATTGTTTTCCTAAAAAAGATGAGTTAGAATTATTATTTTGGAAAGTGCTAAATGATTTAGAAAATTTAATCAAATTTTGGCTAGAAAATTAAGAAAATAATACTATTTTTTAAAAAAGAATAAATTAATATTTTTTTTCAAAAAGTAAAATAATATAGATAAAATATTATTTTGATCAAATTTTCTGGCCAAAAAGGTTTGCCTATTATATTTTTAAGAAAGAAAAAAATCTACTTAATTTATGTGTAAATATACATATTGACTATTTATTTAATTTGGTAATGACAAACTTATGTTCAAAATTTCTGTAAAATGTGCTGTAAATTGTTAAATTCTACAGAATTAAATTCTCACCTCATAAGTCAAAATCTCATAATTAACTTACATGTCCAAAATTTCTTGTAAAATATTAAATTCCACAAAATTAAATTCATAACACATAGGTTATAATGTCATAATTAAATAAGAAATATATTTGATTTTTCAAAGAATATTACGGTTCGAAGTTGAGGTATATCAGTTTTAAAGTCTCTTTTATCATAAAATTATGCTATATATACAATAACGACACATGTAATATATATAATCCCACAAATAAATATAGATCTTGCTCCTACCTTATGAACCATTTCGAAACTCCGAACTTATAAAGATTGAGAAGTTTAATCCGTATCGAATTTACCCTAAAAAAGTTAAAATTCTTCATATAGTGTTACGTGTCAACTTCTTGTCCAAAAGCCAAATGTATATCAAAGGTGATGCAATAAAATAACAGAAATCTTATGTAAGATTTCATCAACACCGTCAATTTTTATTTCAATATAATTAACAATATTTTATGATCGTTGATCTTGATAAAGTAGGTAGCTGTCCAATATTTATTATTATTTATCTTAAATTTAGAAAGGAAAACCAAAAAAAAAAACACAAAGTCACGTGACATTAGTTGTTGAAGCACGTGATCTCTAGCTACCATTCTTTTTCTTTTGGACTTCATATATAAATATATAGTTCATACAACCCCCATGCAACAAAAATACACCAAGACAATTATCACTCTTTCATTCACGTAGTCCTAAACACAAAAAAACCTAGCATATCCACCATTTTTTCCGGCGAAAATG
